# Evaluation of acidogenesis products’ effect on biogas production performed with metagenomics and isotopic approaches

**DOI:** 10.1186/s13068-021-01968-0

**Published:** 2021-05-29

**Authors:** Anna Detman, Michał Bucha, Laura Treu, Aleksandra Chojnacka, Łukasz Pleśniak, Agnieszka Salamon, Ewa Łupikasza, Robert Gromadka, Jan Gawor, Agnieszka Gromadka, Wojciech Drzewicki, Marta Jakubiak, Marek Janiga, Irena Matyasik, Mieczysław K. Błaszczyk, Mariusz Orion Jędrysek, Stefano Campanaro, Anna Sikora

**Affiliations:** 1 Institute of Biochemistry and Biophysics PAS, Warsaw, Poland; 2 Faculty of Earth Sciences, University of Silesia in Katowice, Sosnowiec, Poland; 3 Department of Biology, University of Padova, Padova, Italy; 4 Department of Biochemistry and Microbiology, Institute of Biology, Warsaw, University of Life Sciences, Warsaw, Poland; 5 Institute of Geological Sciences, University of Wroclaw, Wrocław, Poland; 6 Institute of Agricultural and Food Biotechnology, Warsaw, Poland; 7 Oil and Gas Institute, National Research Institute, Cracow, Poland

**Keywords:** Acetogenesis, Methanogenesis, Short-chain fatty acids, Biogas, Metagenomics, MAGs, Isotopic analysis, Pathways of methanogenesis

## Abstract

**Background:**

During the acetogenic step of anaerobic digestion, the products of acidogenesis are oxidized to substrates for methanogenesis: hydrogen, carbon dioxide and acetate. Acetogenesis and methanogenesis are highly interconnected processes due to the syntrophic associations between acetogenic bacteria and hydrogenotrophic methanogens, allowing the whole process to become thermodynamically favorable. The aim of this study is to determine the influence of the dominant acidic products on the metabolic pathways of methane formation and to find a core microbiome and substrate-specific species in a mixed biogas-producing system.

**Results:**

Four methane-producing microbial communities were fed with artificial media having one dominant component, respectively, lactate, butyrate, propionate and acetate, for 896 days in 3.5-L Up-flow Anaerobic Sludge Blanket (UASB) bioreactors. All the microbial communities showed moderately different methane production and utilization of the substrates. Analyses of stable carbon isotope composition of the fermentation gas and the substrates showed differences in average values of δ^13^C(CH_4_) and δ^13^C(CO_2_) revealing that acetate and lactate strongly favored the acetotrophic pathway, while butyrate and propionate favored the hydrogenotrophic pathway of methane formation. Genome-centric metagenomic analysis recovered 234 Metagenome Assembled Genomes (MAGs), including 31 archaeal and 203 bacterial species, mostly unknown and uncultivable. MAGs accounted for 54%–67% of the entire microbial community (depending on the bioreactor) and evidenced that the microbiome is extremely complex in terms of the number of species. The core microbiome was composed of *Methanothrix soehngenii* (the most abundant), *Methanoculleus* sp., unknown *Bacteroidales* and *Spirochaetaceae*. Relative abundance analysis of all the samples revealed microbes having substrate preferences. Substrate-specific species were mostly unknown and not predominant in the microbial communities.

**Conclusions:**

In this experimental system, the dominant fermentation products subjected to methanogenesis moderately modified the final effect of bioreactor performance. At the molecular level, a different contribution of acetotrophic and hydrogenotrophic pathways for methane production, a very high level of new species recovered, and a moderate variability in microbial composition depending on substrate availability were evidenced. Propionate was not a factor ceasing methane production. All these findings are relevant because lactate, acetate, propionate and butyrate are the universal products of acidogenesis, regardless of feedstock.

**Supplementary Information:**

The online version contains supplementary material available at 10.1186/s13068-021-01968-0.

## Background

Anaerobic digestion (AD) of biomass to methane and carbon dioxide is a widespread process in the natural environment or it can be the result of human activity. This complex process involves the interaction of many groups of microorganisms responsible for, respectively, hydrolysis of polymeric compounds to monomers, acidic fermentations, acetogenesis and methanogenesis in anaerobic environments where the concentrations of electron acceptors, such as nitrates, compounds of iron (III) and manganese (IV) or sulfates, are low and the redox potential is below –240 mV [1–4]. AD is both a promising method for renewable energy production and a way to treat tons of waste generated around the world. AD can be conducted in one-stage or multi-stage systems where hydrolysis and acidogenesis are separated from acetogenesis and methanogenesis. Multistep systems provide optimal conditions for each step, stabilize the processes, and increase energy recovery from biomass [5, 6].

Due to the limited number of substrates for methanogenesis (acetate, carbon dioxide and hydrogen/formate, methylated compounds), the acetogenic and methanogenic steps of AD are tightly connected, the methanogens and acetogens forming syntrophic systems. The essence of the syntrophic interactions is interspecies electron transfer (IET) making the entire syntrophic metabolism efficient and thermodynamically favorable. IET can occur indirectly, mediated by hydrogen and formate (IIET), or directly (DIET) via contact between the microorganisms [7–10]. The metabolic pathways utilized for syntrophic oxidation of common non-gaseous products of acidogenesis include beta-oxidation for butyrate; the methylmalonyl-CoA pathway recognized in *Syntrophobacter* [11, 12] or the dismutation pathway recognized in *Smithella propionica* for propionate [13]; the Wood–Ljungdahl pathway for acetate; the pathway of ethanol oxidation recognized in the genera *Pelobacter* and *Desulfovibrio* in the absence of other electron acceptors [14]; the lactate oxidation recognized in *Desulfovibrio* in the absence of sulfate [9, 15]. Studies on *Acetobacterium woodii* revealed methanogens-independent metabolic pathways of (i) ethanol and carbon dioxide conversion to acetate involving a bifunctional ethanol/acetaldehyde dehydrogenase [16]; (ii) lactate oxidation to acetate involving a complex composed of the FAD-dependent lactate dehydrogenase (LDH) and the electron transfer flavoprotein (EtfA/B) [17]. Both require the reductive carbon monoxide dehydrogenase/acetyl-CoA synthase pathway. Genes encoding the lactate-oxidizing metabolic machinery homologous to those of *A. woodii* and *D. vulgaris* are present in the domain *Bacteria*[18]. The *Syntrophomonadaceae* are highly specialized syntrophic microbes that can oxidize butyric, propionic and long-chain fatty acids, with the best recognized species being the butyrate-oxidizing *Syntrophomonas wolfei* and the propionate-oxidizing *Syntrophobacter fumaroxidans, S. wolinii, S. pfennigii, S. sulfatireducens*[19–22]. Another butyrate oxidizer is *Syntrophus acidotrophicus* (*Syntrophobacterales* order in *Deltaproteobacteria*), whereas representatives of the *Desulfotomaculum* and *Pelotomaculum* genera from the *Peptococcaceae* family (*Clostridiales*) [23] or *Smithella propionica* (*Syntrophobacterales* order in *Deltaproteobacteria*) [24] are recognized propionate oxidizers. Known acetate-oxidizing bacteria belong to the following groups: *Synergistetes*—genera *Synergistes* [25]; *Clostridia*—*Thermoacetogenium phaeum*, *Clostridium ultunense, Clostridium sporomusa* and *Moorella* sp.; and the *Deltaproteobacteria*—*Geobacter* spp. [7]; *Spirochaetes* [26]. Uncultivable *Cloacimonetes*, including WWE1 (Waste Water of Evry 1), are capable of propionate and butyrate oxidation [9, 27]. Representatives of *Chloroflexi* and *Plantomycetes* are probably also involved in butyrate oxidation [20]. Previously, methane-producing *Archaea* were thought to belong only to the *Euryarchaeota* (orders: *Methanobacteriales, Methanococcales, Methanomicrobiales, Methanosarcinales, Methanopyrales*, *Methanocellales* and *Methanomassiliicoccales*) [28–31], but recent studies indicate that they may also belong to *Bathyarchaeota* and *Verstaraeteachaeota* [32–36]. It is noteworthy that many *Archaea* and most of the *Bacteria* belong to the group of "viable but non-culturable" (VBNC) microorganisms, and new species are detected on the basis of data obtained from metagenomic studies of environmental or anaerobic digesters’ samples [37]. Methane is a by-product in the reaction of methyl coenzyme M with coenzyme B, catalyzed by archaeal methyl coenzyme M reductase (encoded by the *mtrA* gene) regardless of the methanogenic pathways, *i.e.,* splitting of acetate (acetoclastic/acetotrophic methanogenesis); reduction of CO_2_ with H_2_ or formate and, rarely, ethanol or secondary alcohols as electron donors (hydrogenotrophic methanogenesis); reduction of methyl groups of methylated compounds such as methanol, methylated amines or methylated sulfides (hydrogen-dependent and hydrogen-independent methylotrophic methanogenesis) [38, 39]. Surprisingly, only two known genera *Methanosarcina* and *Methanotrix*, formerly *Methanosaeta*, members of the order *Methanosarcinales*, are capable of methane production from acetate. However, the recent studies change the commonly accepted viewpoint that *Methanothrix* species can only utilize acetate as substrate via the acetoclastic pathway. It was shown that the ethanol-fed methanogenic community was dominated by the *Methanotrix* species which were metabolically active via the carbon dioxide reductive (hydrogenotrophic) pathway, rather than the acetate decarboxylation (acetotrophic) pathway*. Methanothrix* species are capable of accepting electrons via the direct interspecies electron transfer (DIET) for the reduction of carbon dioxide to methane [40, 41]. The identified methylotrophic methanogens belong to the *Methanosarcinales* order. All the other known methanogens produce methane by reduction of CO_2_ [2, 4, 28, 29, 39].

Even though the general scheme of anaerobic digestion is commonly known, it is still not completely characterized at the molecular level. Our understanding of the microbial ecology and physiology associated with AD is crucial for process stabilization and optimization. Since the majority of microorganisms involved in AD are not cultivable, the culture-dependent techniques are insufficient. Thus amplicon-based approaches (e.g., 16S rRNA gene sequencing), metagenomics, and genome-centric techniques were employed to analyze samples from different-scale bioreactors [37, 42–44]. The former concentrate on sequencing of the genes encoding 16S rRNA or other selected genes, e.g., the *mcrA* gene, while metagenomics and genome-centric techniques involve shotgun sequencing of total DNA. All these approaches confirmed that the majority of the species involved were not isolated but can be classified at the genus, family or order level performing a taxonomic assignment of the genes/protein encoded [42, 45–50]. Furthermore, the genome-centric approaches allowed obtaining large numbers of metagenome-assembled genomes (MAGs) from anaerobic digesters. In their majority they represent novel microorganisms residing in AD microbiomes whose physiology and potential functions are being recognized. In fact, the use of approaches based on functional prediction and pathways identification allow to putatively assign these species roles in the degradation of organic matter.

To understand metabolic networks in AD microbiomes, the metagenomic studies focus on analysis of the microbiomes’ composition, description of interactions within microbial communities, and assignment of functions in AD steps to specific groups of microbes [37, 51–55]. Metagenomic approaches show interdependencies between microbial communities and the type of feedstock, the C:N ratio, the operating conditions such as temperature, pH, bioreactor construction, organics loading rate, hydraulic retention time, etc. All the factors select and shape the structure of microbiomes, determine the metabolic pathway of methane production in biogas reactors, and influence the efficiency of methane production. It is believed that feedstock is a factor of special significance and it has a great influence on species abundance and on their functional activity [45–50, 56, 57].

Although the studies mentioned above explored the influence of feedstock, it is not completely understood how short-chain fatty acids can influence the last step of organic matter conversion to methane. The aim of the present study is to determine the influence of the common products of the acidogenic step (i.e., butyrate, propionate, lactate and acetate) on the metabolic pathways of methane formation and the microbial community composition. In accordance with the current trends, we combined three independent approaches: metagenomics, stable carbon isotope analysis of the fermentation gas, and monitoring of performance of Up-flow Anaerobic Sludge Blanket (UASB) bioreactors, to describe the complexity of acetogenesis and methanogenesis.

## Methods

### Object of the study

**Fig. 1:**
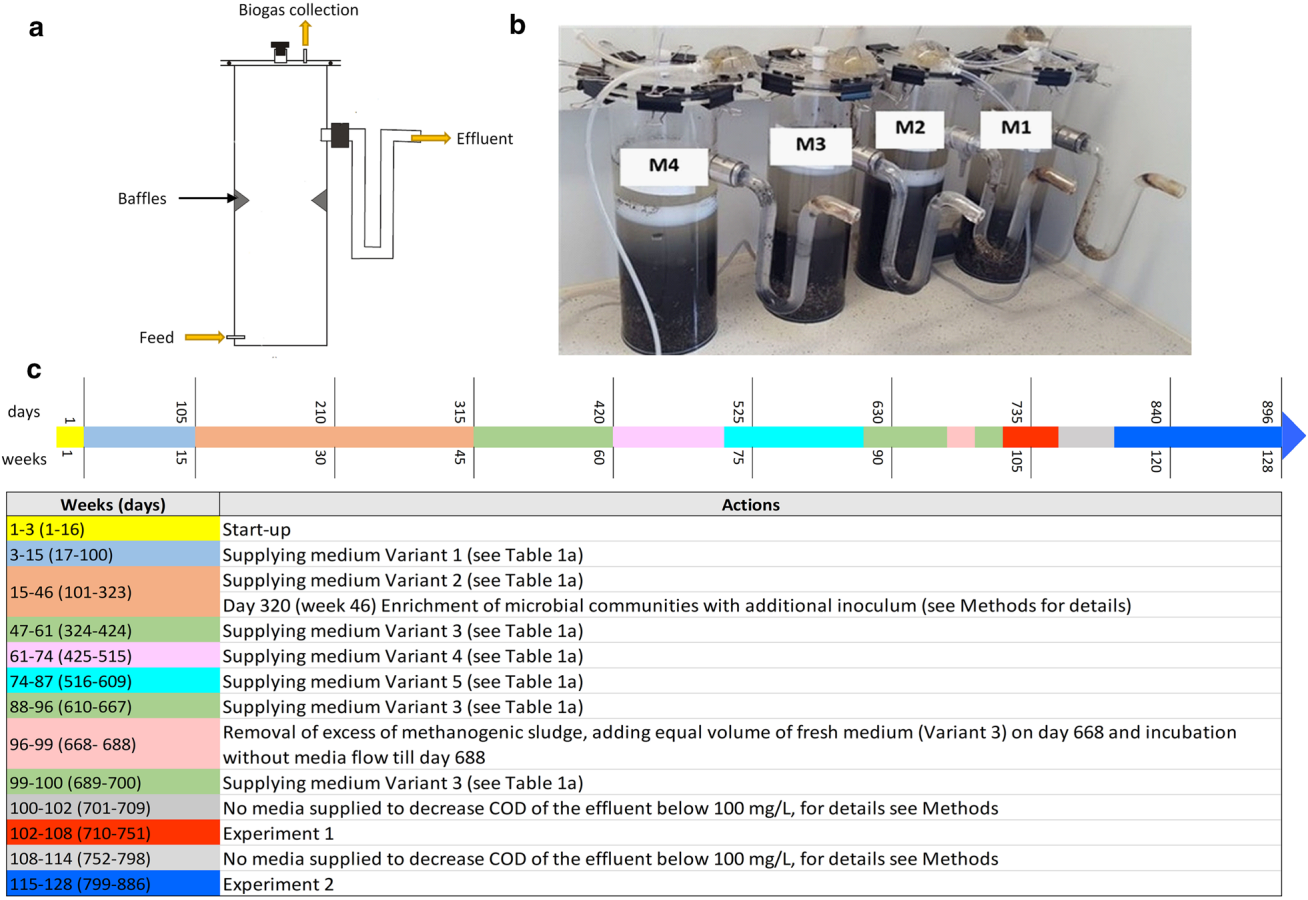
Object of the study and the run of the bioreactors: **a** scheme of UASB bioreactor, **b** four methane-yielding microbial communities in UASB bioreactors, **c** timeline and specific activities done during bioreactors’ operation

The objects of this study were four methane-yielding microbial communities designated M1, M2, M3, M4 in 3.5-L Up-flow Anaerobic Sludge Blanket (UASB) bioreactors (Fig. 1a, b) operating for 128 weeks (896 days) and processing artificial media intended to imitate a mixture of non-gaseous products of different types of acid fermentations with the domination of one of lactate (M1), butyrate (M2), propionate (M3) or acetate (M4). The methanogenic inoculum was anaerobic sludge from a municipal waste treatment plant “Warszawa Południe” in Warsaw, Poland, sampled during the winter (pH = 7.4). The artificial media were based on a modified M9 medium (BD Diffco) [58] without MgSO_4_, CaCl_2_ and glucose (Table 1). The medium was supplemented with the Bacto yeast extract (BD), sodium lactate (VWR Chemicals), butyric acid (Sigma Aldrich)/sodium butyrate (Alfa Aesar), propionic acid (Sigma Aldrich)/sodium propionate (Alfa Aesar), and acetic acid (VWR Chemicals)/sodium acetate trihydrate (Chempur) as shown in Table 1. The concentration of nitrogen-containing compounds in the media were comparable to those in the studies where co-cultures of acetogens and methanogens were examined [59, 60]. At the beginning, all the UASB bioreactors were filled with 1.5 L of the methanogenic inoculum and 2 L of the respective artificial medium (Variant 1) (pH = 7), and incubated for two weeks at room temperature (20–25 °C). Until the 100th day of bioreactors’ operation (week 15), the pH was adjusted with calcium hydroxide (5 g/L). Starting from the 17th day (week 3), the respective media (Fig. 1c, Table 1) were supplied to the UASB bioreactors using a peristaltic pump (ZALIMP, Poland) such that the hydraulic retention time was 7 days. In the 320th day of the process (week 46), 500 mL of the microbial community (anaerobic sludge) was removed and supplemented by methane-yielding sludge from a 50-L UASB bioreactor processing an acidic effluent from molasses fermentation [3] originally inoculated with the same above-described anaerobic sludge from the municipal waste treatment plant “Warszawa Południe” in Warsaw.

**Table 1:** Media composition during bioreactors’ operation

a. Media composition tested till the beginning of Experiment 1 as shown in Fig. 1c					
Component	Medium variant				
	Variant 1	Variant 2	Variant 3 *	Variant 4	Variant 5
Na_2_HPO_4_ (g/L)	6.78	6.78	1.7	0.68	1.36
KH_2_PO_4_ (g/L)	3.00	3.00	0.75	0.30	0.50
NaCl (g/L)	0.50	0.50	0.13	0.05	0.10
NH_4_Cl (g/L)	1.00	1.00	0.25	0.10	0.20
Yeast extract (g/L)	–	2.00	0.50	0.50	0.50
Substrates	Mixture of short-chain fatty acids and their sodium salts as described for Experiment 1, see part b of the Table				
pH adjustment	+	−	−	−	−
Final pH	7.1–7.3	6.0–6.3	5.0–5.6	4.8–5.5	4.9–5.5

b. Media composition during Experiments 1 and 2 (pH values shown in Table 2)								
Bioreactor	M1—lactate		M2—butyrate		M3—propionate		M4—acetate	
Substrates in Medium Variant 3								
**Experiment**	**1**	**2**	**1**	**2**	**1**	**2**	**1**	**2**
Sodium lactate (g/L)	10.71	15.30	1.30	–	1.30	–	1.30	–
Sodium butyrate (g/L)	1.30	–	9.00	12.80	–	–	–	–
Sodium propionate (g/L)	–	–	–	–	9.00	12.80	–	–
Sodium acetate trihydrate (g/ L)	–	–	–	–	–	–	9.80	17.00
Butyric acid (g/L)	–	–	–	–	0.94	–	0.94	–
Propionic acid (g/L)	0.96	–	0.96	–	–	–	0.96	–
Acetic acid (g/L)	0.96	–	0.96	–	1.00	–	–	–

*Variant 3 was supplied from the 610th day (week 88) to the end of bioreactors’ operation

An initial operation period of 667 days (96 weeks) was aimed at adaptation to the substrates, stabilization and optimization of the process, elaboration of optimal concentration of mineral salts and yeast extract in the media, and as a result obtaining substrate-specific microbial communities. The idea of long-term operation of the process was based on (i) studies showing long-term systems producing methane [61, 62]; and (ii) natural and built anaerobic environments with permanent, stable methane production.

From the 610th day (week 88) to the end of bioreactors’ operation, the supplied medium contained 0.5 g/L of yeast extract and Na_2_HPO_4_, KH_2_PO_4_, NaCl and NH_4_Cl (Variant 3 in Table 1a) in amounts corresponding to a fourfold dilution of mineral compounds in the M9 medium. From the beginning till the 701st day (week 101) of bioreactors’ operation, the media contained lactate, propionate, acetate and butyrate with a 70% predominance of one of them (Fig. 1c, Table 1).

Starting from the 701st day of bioreactors’ operation, two main experiments (Experiment 1 and Experiment 2) were carried out. Experiment 1 was preceded by a 9-day period (from the 701st to the 709th days, week 102), during which the medium flow was switched off, and the COD of the effluent from bioreactors dropped below 100 mgO_2_/L, with the aim to minimize the concentrations of organic components with an unknown ^13^C isotope content. In the Experiment 1 (from the 710th to the 751st days, weeks 102–108), the bioreactors were again supplied with the media containing 70% of sodium salts of lactic (M1), butyric (M2), propionic (M3) or acetic (M4) acids (Table 1b). Carbon isotopic compositions of the media were determined (as described in the section “Isotopic analyses”). Between Experiment 1 and Experiment 2, there was a 47-day period (from the 752nd to the 798th days, weeks 108–114) when again the medium flow was switched off to decrease the COD of the effluent from the bioreactors to a value below 100 mgO_2_/L. In the Experiment 2 (from the 799th to the 886th days, weeks 115–127), the media contained only one, previously dominant component, *i.e.*, sodium lactate (M1), sodium butyrate (M2), sodium propionate (M3), and sodium acetate (M4) (Table 1b). Carbon isotopic compositions of the media were again determined. Both experiments ended with collecting of methanogenic sludge samples for DNA analyses on the 751st day (week 108) for Experiment 1 and on the 884th day (week 127) for Experiment 2.

### Analytical methods

During the entire operation, physicochemical parameters (pH, COD—chemical oxygen demand, biogas production rate, CH_4_ and CO_2_ content) describing the UASB bioreactor performance were measured. Additionally, in the periods of Experiments 1 and 2, samples of biogas and effluents from bioreactors were collected for stable carbon isotope analyses (see the section “Isotopic analyses”) and to measure the concentrations of short fatty acids, sulfide (S^2–^), total nitrogen and soluble iron (Fe^2+/3+^) in the effluent from the bioreactors.

The pH of the media and the effluents from the UASB bioreactors as well as the redox potential inside the bioreactors was measured using a standard pH meter (ELMETRON model CP-502) equipped with a combination ORP (redox, mV) electrode type ERPt-13. The chemical oxygen demand (COD) values of the media and the effluents were determined using a NANOCOLOR COD 1500 kit (Macherey–Nagel) according to ISO 15705:2002.

The total rate of gas production was measured using a MGC-1 MilliGascounter (RITTER). The measurement data were recorded manually. The composition of the fermentation gas was analyzed using a HPR20 mass spectrometer (Hiden, England) with a QGA version 1.37 and a Fisons Gas Chromatograph 8000 series with a thermal conductivity detector.

Short-chain fatty acids were analyzed by HPLC with photometric detection (Waters HPLC system with Waters 2996—Photodiode Array Detector, and 300 × 7.8 mm Aminex HPX-87 H column with guard column at 30 °C). The samples were eluted for 45 min with an isocratic flow (0.6 mL/min) of 4 mM sulfuric acid.

The concentration of sulfide (S^2–^) in the effluents was determined using a NANOCOLOR SULFID 3 kit (test No. 985 073, Macherey–Nagel) according to DIN 38405-D26/27. The concentration of total nitrogen in the effluents was determined using a NANOCOLOR TN_b_ 60 kit (test No. 985 092, Macherey–Nagel) based on ISO 7890-1. The concentration of soluble iron (Fe^2+/3+^) in the effluents was determined using a NANOCOLOR IRON 3 kit (test No. 985 037, Macherey–Nagel). Effluents were centrifuged before the analyses to remove microbial cells and debris.

Data from all analyses performed on samples collected from the UASB reactors are presented as mean values ± SD (standard deviation).

XLSTAT software by Addinsoft was used to prepare the box-plot figures and calculate Kolmogorov–Smirnov and Mann–Whitney tests for detailed presentation of COD reduction and methane production over time and testing similarities between the samples for the periods of Experiments 1 and 2.

### Total DNA isolation and sequencing

Total DNAs from the microbial communities formed in all UASB reactors were isolated from samples of anaerobic sludge taken on the 751st day (week 108) for Experiment 1 and on the 884th day (week 127) for Experiment 2. There were three independent sample collections from across the whole bioreactor using a glass pipette with a broken tip. The samples of the sludge were immediately frozen in liquid nitrogen and, before DNA isolation, they were combined in one tube and mixed. DNA was extracted and purified using a DNeasy PowerSoil kit (Qiagen) in five replicates according to the manufacturer's protocol with some modifications. Five samples (0.3 g each) were placed into five bead tubes for extraction, incubated at 70 °C for 15 min, and shaken horizontally in a MoBio vortex adapter for 15 min at maximum speed. The remaining steps were performed as directed by the manufacturer. DNA quality was checked by running the sample on 1% agarose gel and template quantity was measured by a fluorimeter using Qubit 3.0 and High Sensitivity Picogreen reagents (Thermo, USA). Final samples of DNA extracted from the five replicates were pooled and stored at − 80 °C.

DNA sequencing: DNA was mechanically sheared using Covaris (Covaris, MA, USA) and sequencing libraries were constructed using KAPA Library Preparation kit (KAPA Biosystems, Wilmington, USA). The libraries were quality-checked using KAPA Library Quantification kit (KAPA-Roche, Basel, Switzerland), pooled in equimolar ratio and sequenced on a NextSeq 550 instrument using the NextSeq HighOutput reagent v2.5 (300 cycle) chemistry kit (Illumina, San Diego, USA).

### Metagenomics

Illumina DNA sequences were filtered using Trimmomatic software (v0.39) [63]. Analysis of the shotgun reads was performed to determine the microbial composition using MetaPhlAn3 (v3.0.7–0) [64], while the heat-map visualization was generated with “hclust2.py” software. The relative abundance profiles obtained from MetaPhlAn3 and a Newick tree relating all the species included in MetaPhlAn 3.0 were used to calculate the weighted UniFrac distances with the script “calculate_unifrac.R”. Estimates of microbial community diversity was calculated on the filtered reads using Nonpareil3 software (v3.3.3–1) [65]. Filtered reads were co-assembled with MEGAHIT (v1.2.4-beta) [66]. All the scaffolds shorter than 1 kb were removed from the assembly and assembly statistics were determined using QUAST_1 [67]. N50 and N90 values are statistics of a set of contigs or scaffolds lengths. The N50 value is calculated by first ordering every contig/scaffold by length starting from the longest. Then, starting from the longest contig/scaffold, the length of each is summed until this running sum is equal to one-half (or 90% for N90) of the total length of all contigs/scaffolds in the assembly. Metagenome binning was performed with metabat2 (v2.12) [68]. The quality of the metagenome-assembled genomes (MAGs) was determined using CheckM software (v1.0.3) [69] and evaluated considering the MAG quality standards proposed by the Genomic Standards Consortium [70]. The average nucleotide identity (ANI) was calculated considering the genomes deposited in the NCBI Reference Sequence Database [71]. The putative taxonomic classification of the unclassified MAGs were further assessed based on ubiquitous proteins with GTDB-Tk (v1.0.2) [72]. The genomes hits having ANI higher than 95% threshold were used for MAGs classification at the species level [73, 74]. The number of recovered MAGs reported in the global AD microbiome database [37] was estimated calculating the ANI values with dRep software (v2.3.2) [75]. Genes were predicted for each MAG using Prodigal (v2.6.3) and annotated using a combination of strategies based on eggNOG-mapper (v2.0.1–1) [76], DRAM (v1.1.1–0) [77] and KEGG pathway analysis [78]. MAGs coverage was determined through sequences recruitment using Bowtie 2 (v2.2.4) [79] and checkM (v1.0.3) [69].

Raw sequence data were uploaded to the Sequence Read Archive (SRA, NCBI) with project ID PRJNA680596.

### Analyses of stable carbon isotope composition of fermentation gas and substrates

Samples of the fermentation gases were collected from the UASB bioreactors using a syringe and injected into 20-ml glass ampoules filled with a supersaturated NaCl water solution. Stable carbon isotope analyses of carbon dioxide and methane were carried out with an on-line method on a Delta V Advantage Mass Spectrometer coupled with a Trace GC Ultra gas chromatograph with a GC Isolink device (Thermo Scientific). The GC column used for gas analyses was a HP-PLOT/Q (Agilent Technologies, dimensions: 30 m × 0.32 mm × 20 µm). Helium was used as the carrier gas. The GC oven was initially held at 30 °C for 4 min, then heated at a rate of 10 °C/min to 210 °C, and held for 4 min. A CO_2_ certified gas standard (δ^13^C_VPDB_ = − 36.2‰, Air Liquide Deutschland, GmbH) was used for calibration. A gas with the known carbon isotopic composition was analyzed regularly to check the accuracy of the measurement.

Stable carbon isotope analyses of substrates (ingredients in the fermentation experiment) were carried out using two analytical techniques: an off-line preparative system and continuous flow. The first analytical technique involved the use of about 2–5 mg or µl (where relevant) of pure substrates, which were combusted using a CuO wire in a sealed ca. 10 cm^2^ quartz tube, under vacuum at 900 °C. The produced CO_2_ gas was cryogenically purified off-line (liquid nitrogen and dry ice + ethanol mixture). The purified gas was introduced into an isotope ratio mass spectrometer (IRMS; Delta V Advantage/dual inlet, Thermo Scientific) for the analysis of the stable carbon isotope ratio.

The continuous flow technique was performed with a Thermo Finnigan Elemental Analyzer interfaced via a Conflo IV to a Finnigan Delta V Advantage (EA–IRMS). About 400 µg was weighted and placed into a tin capsule, sealed and packed using a hand-press device. The EA operated at an oxidation furnace temperature of 1020 °C, reduction furnace temperature of 650 °C, and a packed-column temperature of 45 °C [80].

For the normalization of the δ^13^C values, international standards (NBS22, USGS24 and USGS40 distributed by the International Atomic Energy Agency, Vienna) were used and then the values were recalculated and reported relative the Vienna Pee Dee Belemnite (V-PDB) scale with ± 0.1‰ precision [81, 82].

## Results

### Performance of the UASB bioreactors M1–M4

To examine lactate, butyrate, propionate and acetate transformation to methane and carbon dioxide, four methane-producing microbial communities continuously processing, respectively, lactate-, butyrate-, propionate- and acetate-rich artificial media in UASB reactors were studied. Composition of the growth media was designed to imitate the acidic products cocktail of microbial communities in environments where, respectively, lactate, butyrate, propionate and acetate dominate in the acidogenic step of AD. Both the acetogenic and methanogenic steps took place in the bioreactors. Similar to a previous study [18], methane-yielding microbial communities were used instead of pure cultures of microorganisms. Our system is novel and closer to the natural environments and biogas digesters where microbial communities and not pure cultures exist.

**Fig. 2:**
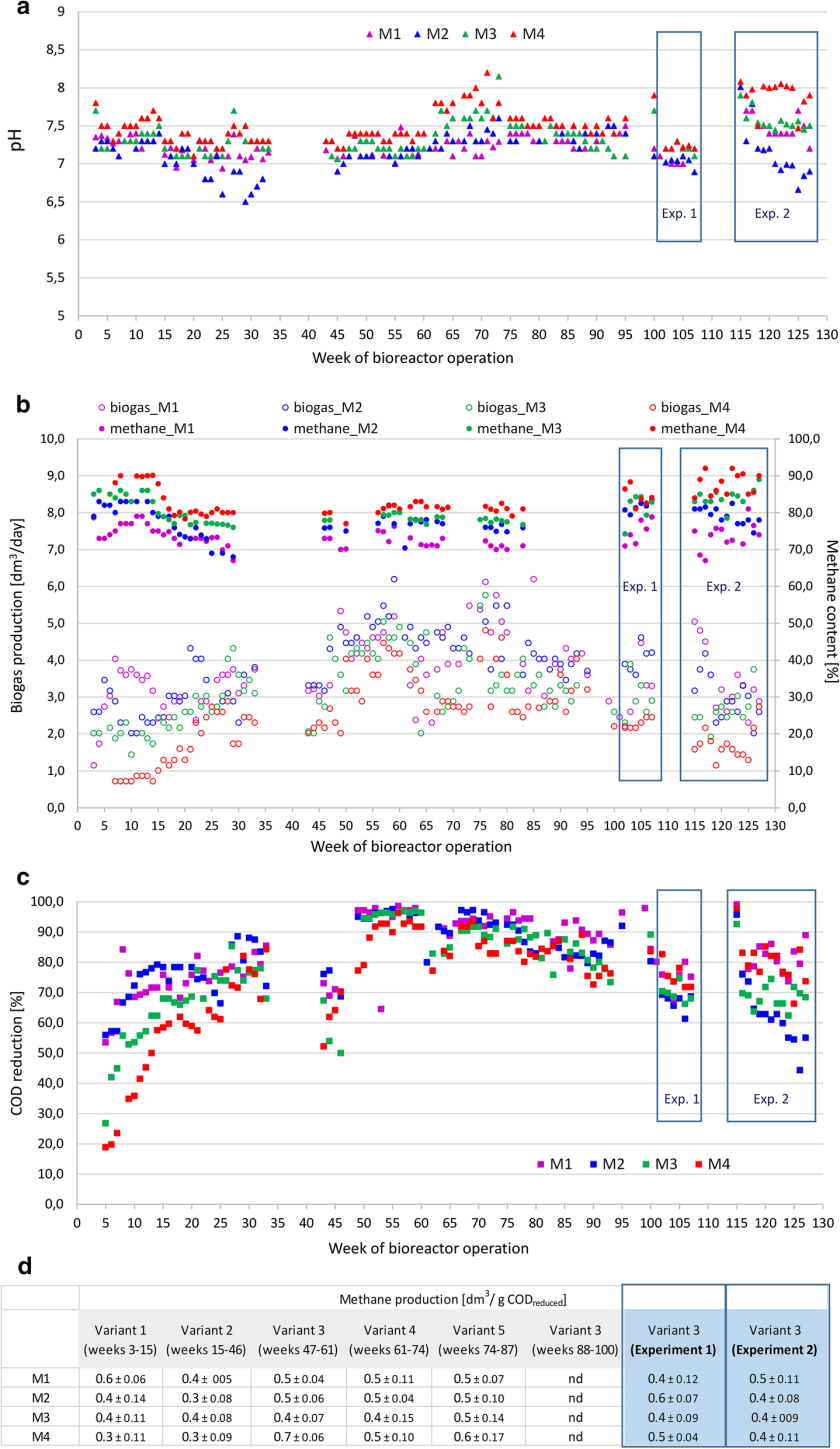
Performance of the bioreactors M1–M4: **a** pH of the effluents, **b** total biogas production per day (left axis) and percentage of methane in biogas (right axis), **c** substrate utilization measured as COD reduction, **d** methane production per g of COD reduction for each medium variant presented in Table 1. Methane production was calculated per g of COD reduction according to the equation: daily biogas production × % of methane in biogas / (COD of the medium —COD of the effluent). The periods of Experiments 1 and 2 are framed in all parts of Fig. 2.

**Fig. 3:**
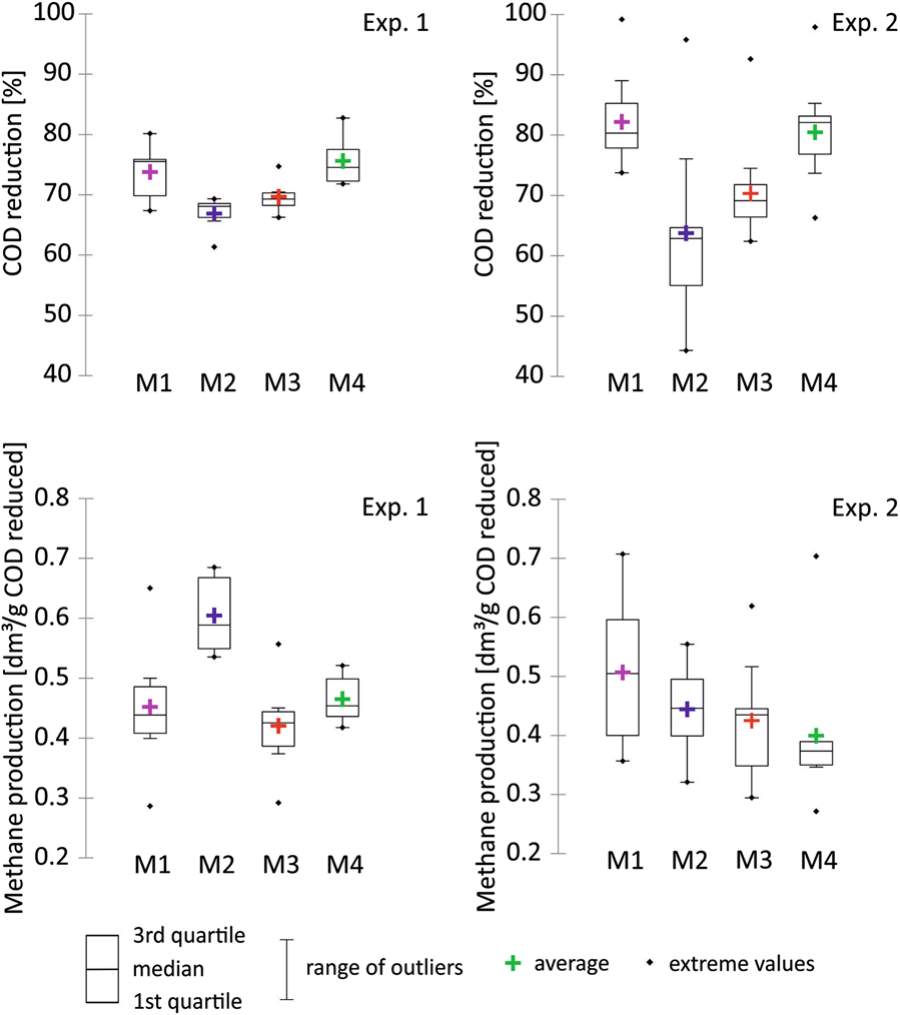
Bioreactors’ performance (COD utilization and methane production) during Experiments 1 and 2. The data were used in the statistical calculations of statistical significance of differences in medians (MWt) and distributions (KSt) between bioreactors performance during Experiments 1 and 2 (see Additional file 2)

**Fig. 4:**
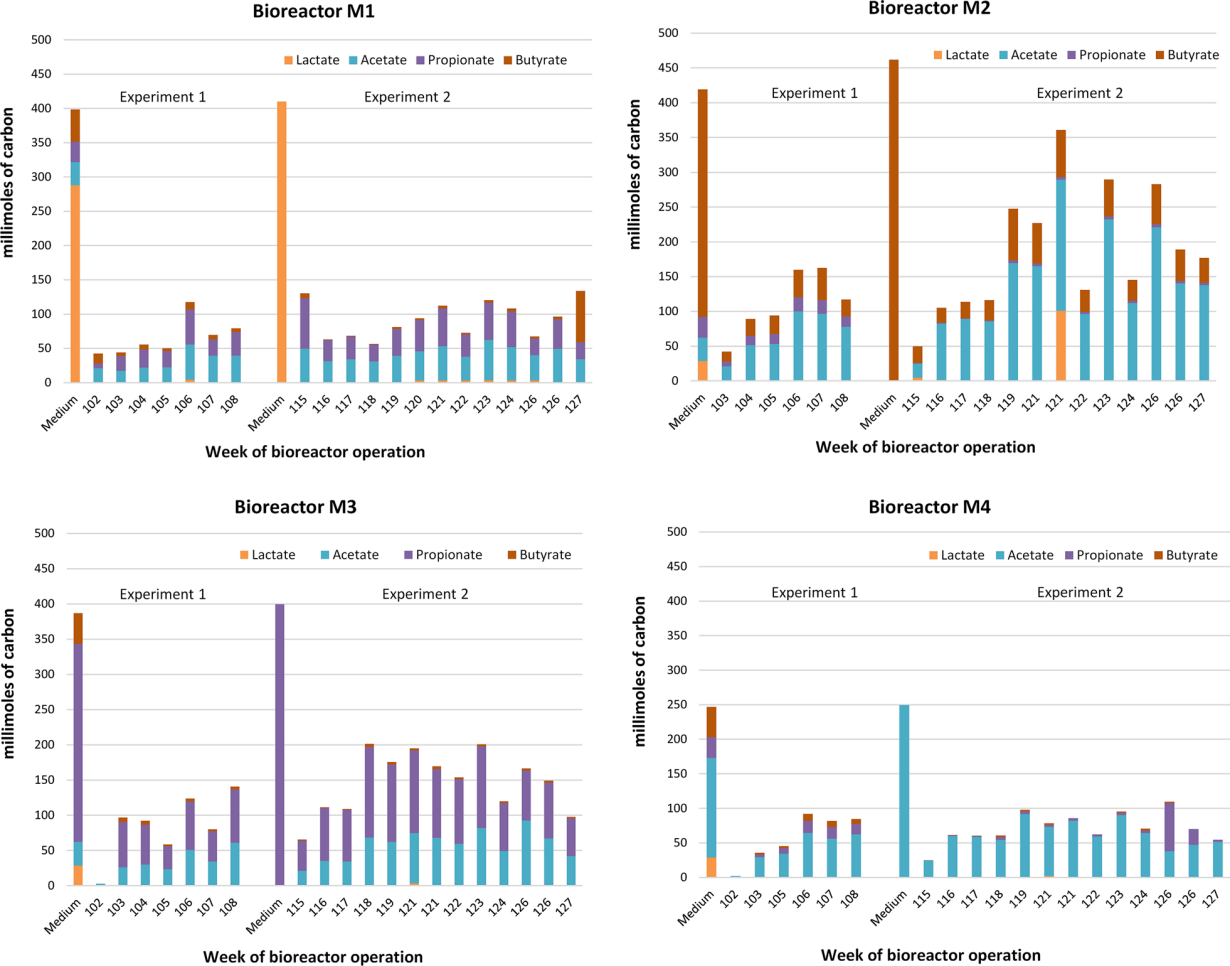
Organic components of the media and effluents from bioreactors expressed in millimoles of carbon at the selected time points of Experiment 1 (weeks 102, 103, 105, 106, 107 and 108, corresponding to days 710–751 of bioreactors operation) and Experiment 2 (weeks 115, 116, 117, 118, 119, 121, 122, 123, 124, 126 and 127, corresponding to days 799–886 of bioreactors operation), M1—domination of lactate, M2—domination of butyrate, M3—domination of propionate, M4—domination of acetate. Collection of methanogenic sludge samples for DNA analyses was done on the 751st day for Experiment 1 (108th week of bioreactor operation) and on the 884th day for Experiment 2 (127th week of bioreactor operation)

Figure 2 and Additional file 1 show the performance (pH of the effluent, daily biogas production, content of methane in biogas, substrate utilization measured as COD reduction, and methane production per g of substrate’s COD reduction) of bioreactors M1–M4 operated for 128 weeks, whereas Figs. 3, 4 and Table 2 report more detailed data from operation of bioreactors M1–M4 in the periods of Experiment 1 and Experiment 2, including the composition of the effluents from the bioreactors.

**Table 2:** Summary of physicochemical parameters characterizing bioreactors operation in Experiments 1 and 2. M1—domination of lactate, M2—domination of butyrate, M3—domination of propionate, M4—domination of acetate

Parameter	COD [g/L]		Substrate utilization [%]	pH		Methane content in biogas [%]	Methane [dm^3^/day]	Methane [dm^3^/g C OD reduced]	S^2-^ [mg/L]		Fe^2+/3+^ [mg/L]		Total nitrogen [mg/L]		Redox potential [mV]
Bioreactor	Medium	Effluent		Medium	Effluent				Medium	Effluent	Medium	Effluent	Medium	Effluent	
**Experiment 1**															
M1	14.1	3.7±0.7 (3.5)	73.8±5.0 (75.2)	5.00	7.1±0.1 (7.1)	74.6±3.0 (77.8)	2.3±0.7 (2.6)	0.4±0.1 (0.5)	<0.05*	0.08±0.02 (0.1)	<10*	0.6±0.01 (0.6)	115.0	96.0	− 280
M2	16.3	5.4±0.5 (5.1)	66.9±3.0 (68.7)	5.40	7.0±0.7 (6.9)	81.5±1.4 (83.4)	3.3±0.3 (3.5)	0.6±0.1 (0.6)	<0.05*	0.14±0.05 (0.1)	<10*	1.2±0.2 (1.4)	115.0	108.0	− 255
M3	16.6	5.0±0.5 (5.3)	69.7±2.9 (68.1)	5.50	7.2±0.1 (7.1)	81.3±3.9 (82.8)	2.3±0.5 (2.4)	0.4±0.1 (0.4)	<0.05*	0.14±0.01 (0.1)	<10*	0.9±0.1 (0.9)	115.0	112.0	− 280
M4	11.0	2.7±0.5 (3.1)	75.6±4.2 (71.8)	5.60	7.2±0.1 (7.2)	76.2±4.4 (84.1)	1.9±0.1 (2.1)	0.5±0.0 (0.5)	<0.05*	0.19±0.06 (0.2)	<10*	0.4±0.1 (0.5)	115.0	120.0	− 305
**Experiment 2**															
M1	12.2	2.2±0.8 (1.3)	82.2±6.7 (89.0)	7.00	7.5±0.2 (7.2)	73.9±3.7 (74.0)	2.5±0.6 (2.9)	0.5±0.1 (0.4)	<0.05*	0.08±0.03 (0.1)	<10*	0.8±0.06 (0.8)	115.0	110.0	− 400
M2	16.7	6.1±2.1 (7.5)	63.7±12.6 (55.1)	7.00	7.2±0.4 (6.9)	79.1±2.3 (78.0)	2.4±0.5 (2.0)	0.4±0.1 (0.4)	<0.05*	0.12±0.06 (0.1)	<10*	1.5±0.6 (1.7)	115.0	110.0	− 363
M3	14.9	4.4±1.1 (4.7)	70.3±7.5 (68.5)	7.00	7.6±0.1 (7.5)	84.2±2.4 (89.0)	2.2±0.4 (2.4)	0.4±0.1 (0.5)	<0.05*	0.12±0.06 (0.2)	<10*	0.6±0.1 (0.6)	115.0	90.0	− 333
M4	9.5	1.9±0.7 (2.5)	80.5±7.4 (73.7)	7.20	7.9±0.2 (7.9)	87.8±2.9 (90.0)	1.5±0.4 (2.5)	0.4±0.1 (0.7)	<0.05	0.22±0.21 (0.1)	<10*	0.4±0.1 (0.5)	115.0	115.0	− 343

*The limit of quantification

In parenthesis, there are data from the time of sample collection for DNA isolation (day 751, /week 108/ for Experiment 1 and day 884 /week 127/ for Experiment 2). COD utilization and methane production are shown as arithmetic means ± SD

Analysis of the physicochemical parameters revealed that pH of the effluents from the UASB bioreactors was close to neutral, which indicates stability of the methanogenesis process. Notice that the pH of the supplied media was neutral till the 100th day (week 15) of bioreactors’ operation. From the 101st day (week 15) of bioreactors’ operation till the beginning of Experiment 2, acidic media (pH ≈ 5) were supplied (Table 1a). The pH of the media during Experiment 2 was 7 (Tables 1 and 2). The optimum pH range for methane production is 6.8—7.2 [83]. Thus, two-step or multi-step anaerobic digestion ensures pH stability, which is a commonly described advantage of such systems [5, 6].

Because the concentration of mineral salts in the substrate subjected to methanogenesis influences the methanogenic process, the periods of stabilization and optimization involved supplying undiluted and diluted (four-, ten- and fivefold) mineral compounds of the M9 medium to the bioreactors. Also, different concentrations of the yeast extract (0.5 and 2 g/L) were tested (Table 1a). In all bioreactors, (i) production of biogas containing 70–90% of methane and (ii) substrate utilization measured as % reduction of the COD of substrates were observed (Fig. 2b–d and Table 2). Interestingly, the highest methane content was found in the biogas formed in the bioreactor fed with the substrate rich in acetate (M4), whereas the lowest one in the biogas produced as the result of processing of the substrate rich in lactate. To take into account differences in CODs of the media and to standardize the results of methane generation, we calculated the amount of methane produced per g of COD reduction (Fig. 2d) in different periods of bioreactors’ operation. We concluded that in the experimental system used, no spectacular differences in methane production were observed for the various medium variants shown in Table 1.

Since the periods of Experiments 1 and 2 were the most important for the study, their precise analysis is presented below with reference to Figs. 3, 4 and Table 3. The usage of the substrate during Experiment 1 was on average ~ 70%. However, over time, the analysis revealed that the acetate-containing medium (M4) was utilized more efficiently in comparison to the butyrate- (M2), and propionate-containing (M3) media (*p* = *0.005* and *p* < *0.03*, respectively). Differences in the substrate usage were much more evident during Experiment 2. Lactate- (M1) and acetate-containing (M4) media were utilized at a similar level (on average ~ 80%), whereas lower values were observed for propionate (M3) and butyrate (M4) (~ 70% and ~ 60%, respectively). Over time, the analysis revealed differences between M1 *vs* M2 and M1 *vs* M3 (*p* < *0.001*) as well as between M4 *vs* M2 and M4 *vs* M3 (*p* ≤ *0.001*). Also propionate (M3) was metabolized more efficiently than butyrate (M2), *p* ≤ *0.025* (Table 2, Fig. 3, Additional file 2)*.*

**Table 3:** Isotopic composition of carbon in substrates and products of Experiment 1 and Experiment 2, M1—domination of lactate, M2—domination of butyrate, M3—domination of propionate, M4—domination of acetate

Bioreactor	δ^13^C_SUB_ [‰]	δ^13^C(CH_4_) [‰]	δ^13^C(CO_2_) [‰]	α^13^C_CO2-CH4_
**Experiment 1**				
M1	− 23.0	− 40.3	1.1	1.043
M2	− 26.3	− 46.6	0.6	1.050
M3	− 31.8	− 51.0	− 6.8	1.047
M4	− 45.4	− 53.3	− 20.9	1.034
**Experiment 2**				
M1	− 23.0	− 36.2	4.1	1.042
M2	− 26.3	− 46.1	6.6	1.055
M3	− 31.8	− 57.0	− 5.4	1.054
M4	− 45.4	− 57.2	− 33.4	1.026

Methane contents in the produced biogas during Experiment 1 were on average 75%, 82%, 81% and 76% for M1, M2, M3 and M4, respectively. Methane production was calculated per gram of reduction of the substrate COD. In Experiment 1, the results were on average 0.4 ± 0.1, 0.6 ± 0.1, 0.4 ± 0.1 and 0.5 ± 0.0 dm^3^/g COD reduction for bioreactors M1, M2, M3 and M4, respectively. Interestingly, over time, the analysis revealed that the highest efficiency was achieved for the M2 microbial community processing the butyrate-containing medium (in comparison to M1 and M3, p ≤ *0.03*; in comparison to M4, *p* ≤ *0.005*). It is noteworthy that the butyrate-containing medium was utilized in 60% only. In Experiment 2, when the media contained exclusively sodium lactate (M1), sodium butyrate (M2), sodium propionate (M3) and sodium acetate (M4), the methane contents were on average 74%, 79%, 84% and 88%, respectively. Interestingly, the highest number was observed in the bioreactor (M4) processing exclusively acetate. The results of methane production were on average 0.5 ± 0.1, 0.4 ± 0.1, 0.4 ± 0.1, 0.4 ± 0.1 dm^3^/g COD reduction for bioreactors M1, M2, M3, M4, respectively. In this case, the analysis over time found a statistically significant difference only between the lactate-utilizing (M1) and the acetate-utilizing (M4) microbial communities (*p* < *0.02*) (Table 2, Fig. 3, Additional file 2).

Qualitative and quantitative analyses of the effluents from bioreactors (Fig. 4) showed that lactate was most effectively and almost completely utilized in all bioreactors independent of its initial concentration in the media. The average percent of butyrate and propionate utilization was 88.5 and 72.2, respectively, when they were the dominant components of the media. Butyrate, propionate and lactate were oxidized to acetate. Lactate was also metabolized to propionate (Fig. 4).

In the effluents from bioreactors, the S^2−^ and Fe^2+/3+^ ions were detected in low concentrations of ≤ 0.2 mg/L and ≤ 1.5 mg/L, respectively (Table 2). It indicated that the processes of sulfate and iron reduction were insignificant, due to the low redox potential in bioreactors, as required for methanogenesis processes (Table 2).

### Isotopic analyses

To find differences between the metabolic pathways of transformation of lactate, butyrate, propionate and acetate to methane and carbon dioxide, isotopic analyses were conducted as a method independent of both monitoring of the bioreactor’s performance and metagenomics.

The carbon isotope signatures (δ^13^C_SUB_) of the substrates used in Experiment 1 and Experiment 2 (organic salts: sodium lactate, sodium butyrate, sodium propionate, sodium acetate) were determined prior to incubation (Table 3). The range of δ^13^C_SUB_ values in the substrates used in this study was from − 45.4 to − 23.0‰, which is similar to those in the natural environment [84, 85]. The distribution of carbon isotopes during decomposition of any organic compound is controlled by the isotope signature of the substrate, the isotopic mass balance controlled by the redox processes, and the kinetic isotope effects [86–88]—in this study particularly relating to microbial degradation of organic salts. In the natural environment, the δ^13^C(CH_4_) values typical for acetate fermentation are in the range from − 60 to − 33‰, while for CO_2_ reduction in range from − 110 to − 60‰ (e.g., [87, 89, 90]). Combined analyses of δ^13^C(CH_4_) and δ^13^C(CO_2_) values in biogas from incubation experiments allow to calculate the potential isotopic fractionation factor α^13^C_CO2-CH4_ (e.g., [86, 91, 92]) according to the equation: α^13^C_CO2-CH4_ = (δ^13^C_CO2_ + 1000)/(δ^13^C_CH4_ + 1000) [87, 93]. The values of α^13^C_CO2-CH4_ in range 1.049–1.095 are typical for CO_2_ reduction, 1.039–1.058 are typical for acetate fermentation, and 1.005–1.03 are typical for methane oxidation [87]. Therefore, tracing changes of the fractionation factor α^13^C_CO2-CH4_ in time is a helpful tool to determine the dominant process responsible for the methanogenesis.

**Fig. 5:**
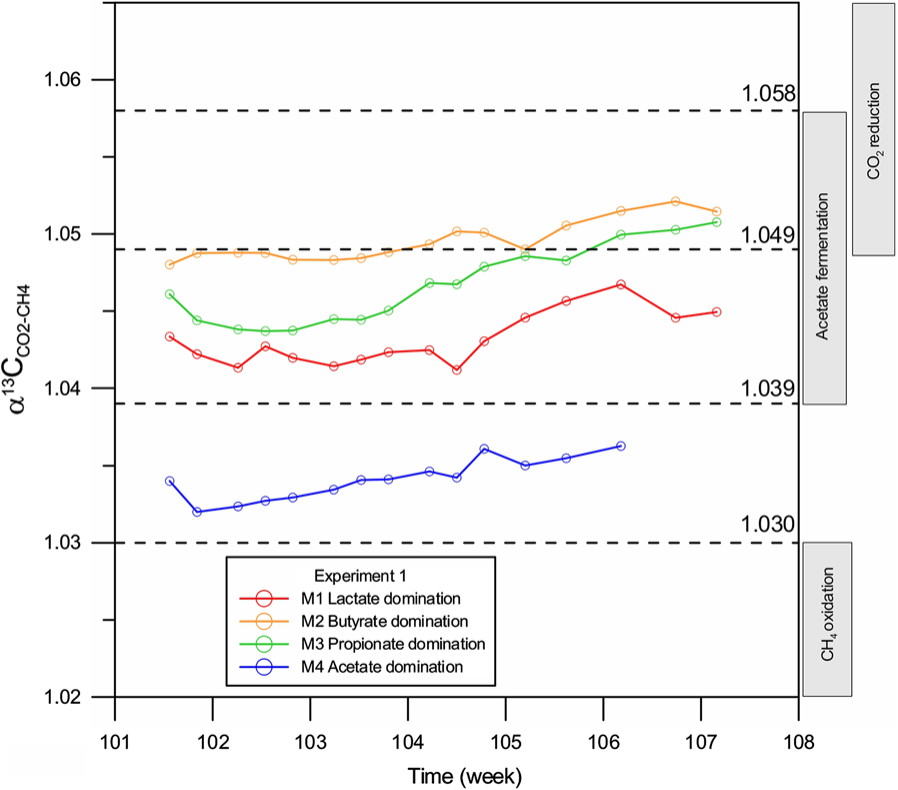
Variation of isotopic fractionation factor α^13^C_CO2-CH4_ in time during Experiment 1

**Fig. 6:**
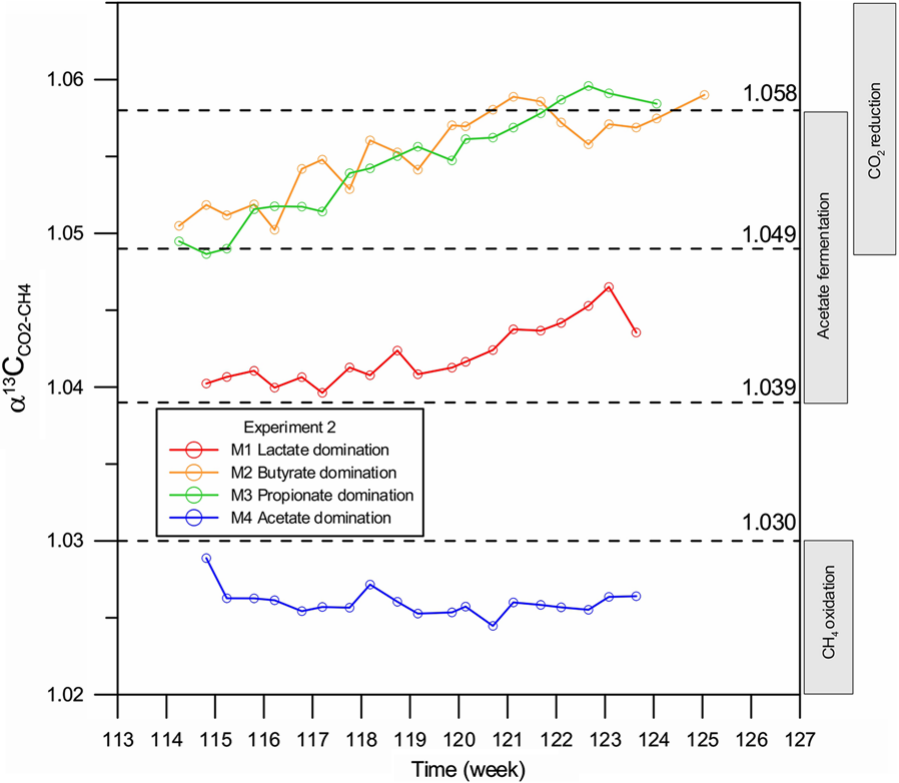
Variation of isotopic fractionation factor α^13^C_CO2-CH4_ in time during Experiment 2

The results of the mean δ^13^C values in CH_4_ and CO_2_ and of the calculated fractionation factor are shown in Table 3. The mean δ^13^C(CH_4_) values obtained for the biogas from the bioreactors were in the range from − 57.2 to − 36.2‰. Such a range of δ^13^C(CH_4_) values is typical for acetate fermentation and partially for mixing of methane from acetate fermentation and CO_2_ reduction**.** In experiments with lactate domination (M1, Experiments 1 and 2), a similar isotopic fractionation of carbon isotopes between CH_4_ and CO_2_ occurred with α^13^C_CO2-CH4_ equalling 1.043 and 1.042, respectively. The lactate was oxidized immediately to acetate (data from HPLC not shown here), which was the direct precursor for CH_4_ and CO_2_ formation. In experiments with butyrate domination (M2, Experiments 1 and 2) and propionate domination (M3, Experiments 1 and 2) we observed an enrichment of light carbon isotopes in CH_4_. The mean δ^13^C(CH_4_) values obtained for the biogas from bioreactors M2 and M3 were in range from − 57.0 to − 46.1‰. The reason for the enrichment of light carbon in CH_4_ is the influence of CO_2_ reduction processes during incubation. This observation is confirmed by the increase of the isotopic fractionation factor α^13^C_CO2-CH4_ in time in cases of butyrate (M2) and propionate (M3) domination, both in Experiment 1 (Fig. 5) and Experiment 2 (Fig. 6).

In the experiments with acetate domination (M4, Experiments 1 and 2), the mean δ^13^C(CH_4_) values were − 53.3 and − 57.2‰, respectively. The δ^13^C(CO_2_) values in the experiments with acetate (M4) differed significantly between Experiment 1 and Experiment 2 and were equal to − 20.9 and − 33.4‰, respectively. The isotopic fractionation factor α^13^C_CO2-CH4_ for incubations with acetate domination (M4) was equal to 1.034 in Experiment 1 and 1.026 in Experiment 2. Such values are close to those typical for acetate fermentation and/or methane oxidation. Processes of methane oxidation were excluded during incubation, because free oxygen was not present in the headspace gas samples (data from gas chromatography not shown here). Calculation of the isotopic mass balance with the equation δ^13^C_calc_ = 0.5 × δ^13^C_CH4_ + 0.5 × δ ^13^C_CO2_ is a very useful tool, especially in the case of the methanogenic decomposition of acetate [94]. Assuming that acetate was the main substrate for the methanogenesis in bioreactor M4, and based on the mean values of δ^13^C(CH_4_) and δ^13^C(CO_2_), the δ^13^C_calc_ value should be similar or close to the δ^13^C value for the sodium acetate used in the experiments. In the case of the experiments with acetate domination (M4, Experiments 1 and 2), the calculated δ^13^C value was equal to − 37.1 and − 45.7‰, respectively. The δ^13^C for the sodium acetate used in this study was − 45.4‰. The calculations of the isotopic mass balance for Experiment 2 confirmed the decomposition of sodium acetate as the only substrate in the bioreactor. The enrichment of light carbon isotopes in the CO_2_ is an effect of the access of the microorganisms to a large pool of readily available acetate, continuously transferred to the bioreactors. The microorganisms decompose the ^13^C-depleted molecules of acetate slightly faster, which is typical for most of biodegradation reactions [86, 87, 89, 95]. In Experiment 1 with domination of acetate (M4), lactate was an additional source for decomposition and, therefore, formation of an additional pool of acetate [18]. The δ^13^C value for the lactate used in this study was equal to -23.0‰ and its decomposition with sodium acetate (δ^13^C = − 45.4‰) resulted in the shift to a heavier values of the mean δ^13^C(CH_4_) in Experiment 1 with acetate domination (M4). The isotopic fractionation factor α^13^C_CO2-CH4_ for Experiment 2 with domination of acetate (M4) apparently might indicate methane oxidation, but it is probably not the case, as the incubation took place in an open system with continuous stable supply of new portions of external acetate to the fermentation solution (Additional file 3).

### Metagenomic analysis

To explain the different contribution of the acetotrophic and hydrogenotrophic pathways of methane production depending on the type of substrate, metagenomic analyses of the microbiomes from M1 to M4 were performed.

Shotgun Illumina sequencing was performed on eight samples (four samples M1–M4 from Experiment 1, four samples M1–M4 from Experiment 2), obtaining from 9.5 to 33.6 Gbases of sequences depending on the experiment. Taxonomic analysis of the shotgun reads (unassembled) was performed to have a global representation of the microbiome composition including the rare components. Weighted UniFrac distances calculation revealed a clear separation between samples supplemented with lactate (M1), and the remaining samples, in particular those supplemented with acetate (M4) (Additional file 4). All the samples were dominated by *Euryarchaeota* (average 79.2% relative abundance), while bacteria were 20.8% on average (Additional file 4); in all the reactors the relative abundance of *Archaea* increased even more in the second period. This behavior was particularly evident in samples supplemented with acetate. In total, eight bacterial phyla were identified, and the most abundant are different in the reactors: *Actinobacteria* are abundant in butyrate M2 (Experiment 1), *Bacteroidetes* in lactate M1 (Experiment 2) and acetate M4 (Experiment 1), *Synergistetes* in butyrate M2 (Experiment 2). Analysis of the shotgun reads cannot provide a high level of detail in defining associations between functional pathways and microbial species. For this reason a genome-centric approach was implemented.

Reads belonging to all the experiments were co-assembled obtaining in total 291,272 contigs larger than 1 kb and having a total size of 1,057 Mbp. Contigs larger than 10 kbp accounted for approximately 41% of the total, evidencing the good quality of the assembly process.

**Fig. 7:**
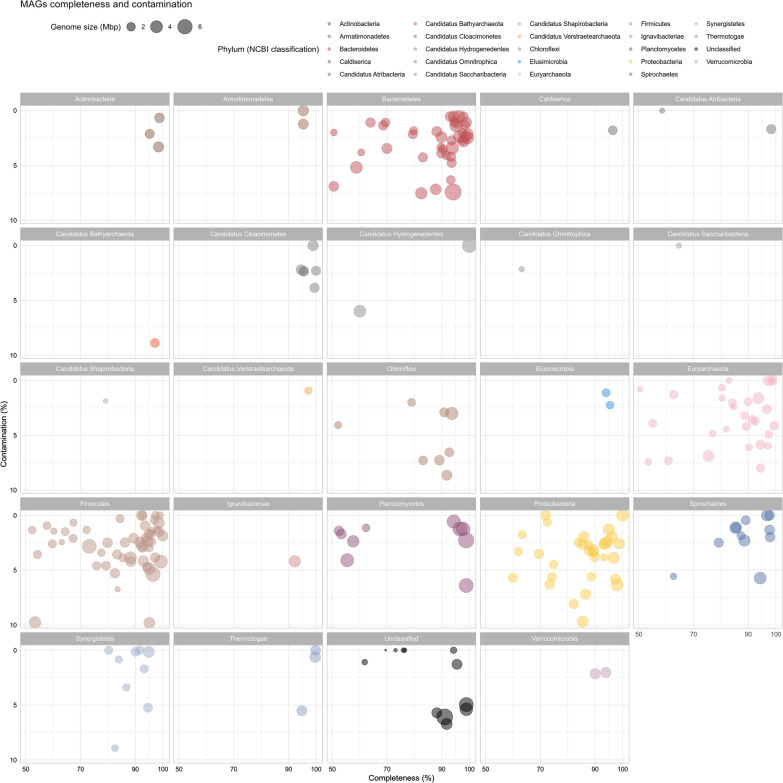
MAGs genome quality. Completeness level and contamination of the 234 MAGs having completeness higher than 50% (Xx axes) and contamination lower than 10% (Yy axes) recovered from the experiment. Colors were assigned according to the taxonomy at phylum level

Binning process recovered 234 Metagenome Assembled Genomes (MAGs) having completeness higher than 50% and contamination lower than 10% (Fig. 7; Additional file 5); 104 of them were of very high quality with completeness higher than 90% and contamination lower than 5%, other 90 MAGs have medium–high quality with completeness higher than 70% and contamination lower than 10%.

**Fig. 8:**
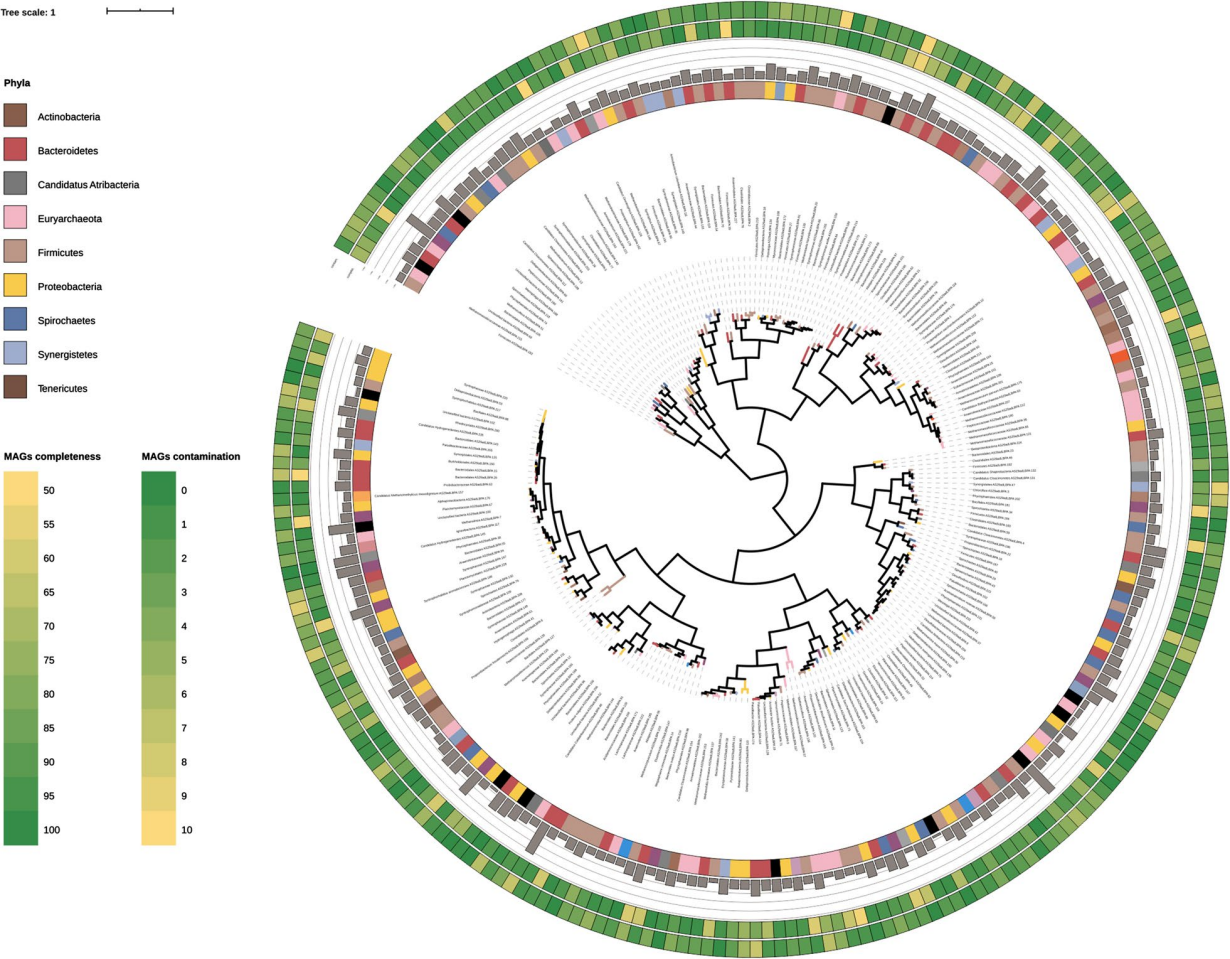
MAGs phylogenetic tree. Phylogenetic tree reconstruction performed according to selected informative proteins. From inside to outside: the phylogenetic tree with the 234 MAGs colored according to their taxonomic assignment (inner circle), the genome size (bar plot in the second circle), MAGs completeness (third circle), contamination (fourth circle), relative abundance in the different experiments depicted as a heat-map with colors ranging from blue (rare MAGs) to red (abundant MAGs)

Taxonomy assignment of MAGs was performed taking into account the results from taxonomic informative proteins, and also Average Nucleotide Identity with the genomes present in NCBI database (Fig. 8; Additional file 6); these analyses revealed the presence of 31 archaeal (13.2%) and 203 bacterial species (86.8%). The high number of *Archaea* is an interesting feature of this metagenomic analysis project.

Reads alignment on the assembled scaffolds highlighted that MAGs accounted for 54–67% of the entire microbial community (depending on the experiment) and evidenced that the microbiome is extremely complex in terms of species number. It was not really expected that a microbiome focused on the last part of the AD food chain was so complex. The metric of sequence diversity calculated on the shotgun not-assembled reads ranged from 18.1 (bioreactor M3, Experiment 2) to 19.8 (bioreactor M2, Experiment 1) and evidenced that the complexity of the AD microbiome is similar to that calculated for human stool samples [65]. The values for all the samples recovered from the first sampling point were higher than the corresponding values for the second sampling point; this is expected since a simplification of the feedstock resulted in a reduction of the number of species present.

**Fig. 9:**
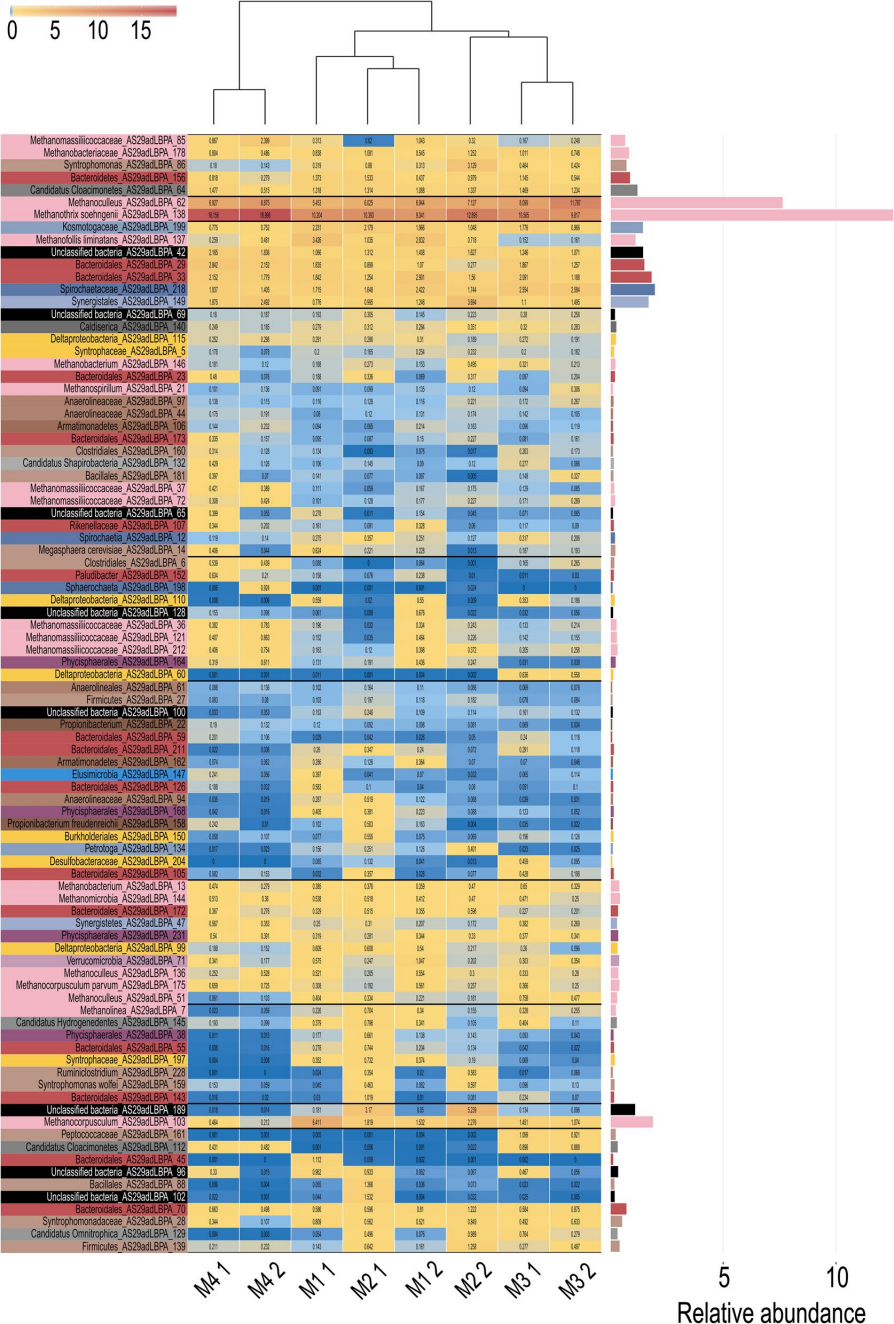
MAGs relative abundance. Relative abundance of MAGs represented as a heat-map with colors ranging from blue (rare MAGs) to red (most abundant MAGs). Colors reported in the left part are according to the taxonomy at phylum level. The tree shown in the top part of the figure represents the distance among coverage profiles. The bar plot on the right shows the average relative abundance of the MAGs measured in the different experiments. M1_1 and M1_2 denote bioreactor M1 Experiment 1 and 2, respectively; M2_1 and M2_2 denote bioreactor M2 Experiment 1 and 2, respectively; M3_1 and M3_2 denote bioreactor M3 Experiment 1 and 2, respectively; M4_1 and M4_2 denote bioreactor M4 Experiment 1 and 2, respectively;

By taking into account MAGs relative abundance for each experiment, only 8–15 of them (depending on the sample considered) had an abundance higher than 1% (Fig. 9; Additional file 7). Interestingly, five MAGs were identified at high abundance in all the samples (MAG_138, MAG_62, MAG_42, MAG_33, MAG_218). Considering the average value in all the experiments, MAG_138 (*Methanothrix soehngenii* AS29adLBPA_138) was the most abundant (12.1%) and reached the highest relative abundance in the reactor fed with acetate (18–19%); this archaeal species is highly similar to *Methanothrix soehngenii* GP6 (Average Nucleotide Identity-ANI 99%) an acetoclastic methanogen previously named *Methanosaeta concilii* GP6 (Additional file 6). Two archaeal species were also found with a high average abundance, MAG_62 (7.58%) (*Methanoculleus* sp. AS29adLBPA_62, 96.9% ANI) and MAG_103 (2.03%) (*Methanocorpusculum* sp. AS29adLBPA_103). *Methanoculleus* sp. AS29adLBPA_62 reached a very high abundance when propionate was used as feeding, but it was present at high abundance in all the samples. *Methanocorpusculum* sp. AS29adLBPA_103 had a more “scattered” distribution since it was more abundant in samples from bioreactor M1 (domination of lactate, Experiment 1) and bioreactor M2 (domination of butyrate, Experiment 2).

Considering bacterial species, the most abundant was MAG_218 (2.15%), an unknown species of the *Spirochaetacea*e family (*Spirochaetaceae* sp. AS29adLBPA_218), followed by MAG_33 (1.92%) (*Bacteroidales* sp. AS29adLBPA_33) which is highly similar to *Lentimicrobium* sp. 002433025 (98.52% ANI) and by MAG_149 (1.64%) (*Synergistales* sp. AS29adLBPA_149) belonging to the *Thermovirgaceae* family. MAG AS29adLBPA_218 reached the highest abundance in reactors fed with propionate and in the one fed with pure lactate, while MAG AS29adLBPA_149 was abundant in the reactors fed with pure acetate and butyrate.

### Substrate-specific microbial species

**Fig. 10:**
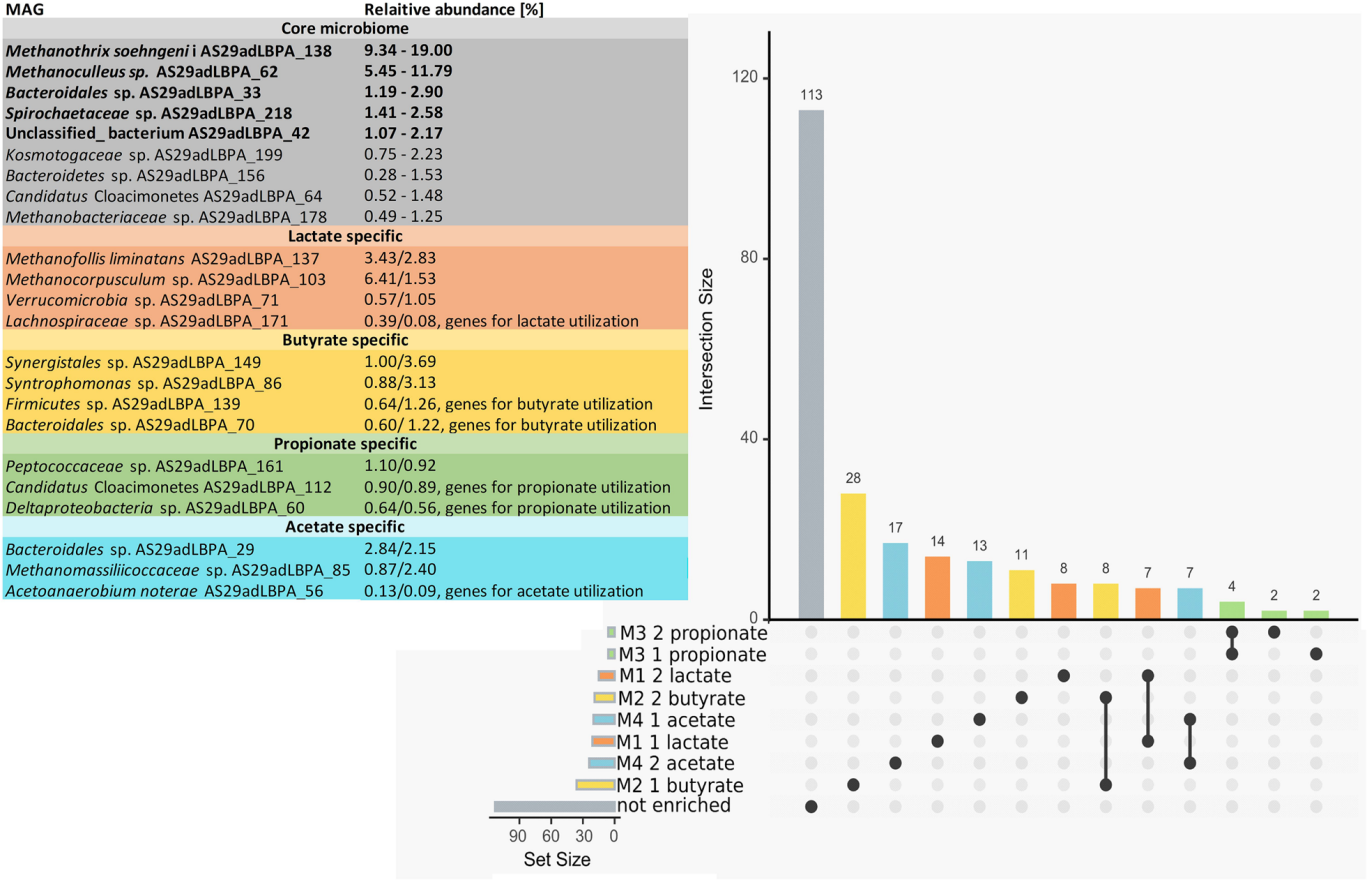
The number of “substrate-specific” MAGs and those having similar abundance in different samples (the core microbiome) are reported. Bars representing substrate-specific MAGs are colored yellow, blue, green and orange, and those representing the core microbiome are shown in gray

The average relative abundance was calculated for all the MAGs and the values obtained for each sample/condition were compared with it (Additional file 7). This revealed the changes in abundance of each species in different samples and helped to identify microbes having substrate preferences. Due to the difficulty in defining preferences for substrate utilization for the MAGs, those specifically enriched in samples fed with one substrate were selected. It was found that 37 MAGs were enriched when acetate was used as feeding (> 1%, *Bacteroidales* sp. AS29adLBPA_29 and *Methanomassiliicoccaceae* sp. AS29adLBPA_85); 47 were enriched on butyrate (> 1%, *Bacteroidales* sp. AS29adLBPA_70, *Firmicutes* sp. AS29adLBPA_139, *Synergistales* sp. AS29adLBPA_149, *Syntrophomonas* sp. AS29adLBPA_86), 8 on propionate (> 1%, *Peptococcaceae* sp. AS29adLBPA_161), and 29 on lactate (> 1%, *Methanofollis liminatans* AS29adLBPA_137; *Verrucomicrobia* sp. AS29adLBPA_71; *Methanocorpusculum* sp. AS29adLBPA_103) (Fig. 10, Additional file 7). These microbes are to a certain extent “specialized” since they have preferences for one substrate; however, in most cases, their relative abundance is low, below 1%. Based on these findings, it seems that butyrate and acetate are the feeding substrates favoring more the growth of substrate-specific MAGs. On the contrary, 113 MAGs did not have preferences for one substrate and they had a more “generalistic” behavior and, thus, can be regarded as a core microbiome (Fig. 10, Additional file 7).

To identify the microbial species, for which the increased abundance in specific reactors is due to their ability to utilize or degrade specific compounds, the coverage profile of MAGs was compared with their predicted functional role, which was determined using a combination of predictive methods (including analysis of KEGG pathways).

#### Butyrate

Metabolic pathways present in the MAGs enriched in reactors fed with butyrate revealed that ten microbes have a complete pathway involved in SCFA and alcohol conversions “Butyrate, pt 1” (Additional file 8)**.** Among these species, two were present at very high abundance in the microbiome: *Synergistales* sp. AS29adLBPA_149 and *Bacteroidales* sp. AS29adLBPA_70. The first one is a high-quality MAG (91.5% compl., 0.0% cont.) reaching 3.7% relative abundance when butyrate was the only carbon substrate provided. It has 21 complete KEGG modules including “fatty acid biosynthesis, elongation” (M00083) and “beta-Oxidation, acyl-CoA synthesis” (M00086) (Additional file 8). It is able to use butyrate catalyzing the conversion with butyrate kinase (K00929) and phosphate butyryltransferase (K00634). The second MAG has very similar characteristics (94.5% compl., 1.0% cont.) but the relative abundance with butyrate as feeding was slightly lower (1.2%). This MAG had a higher number of complete KEGG modules (N = 37) than *Synergistales* sp. AS29adLBPA_149, and this is probably due to its larger genome size (2.56 Mbp in comparison to 2.04 Mbp). These characteristics, suggestive of a more complex metabolism, still included genes for butyrate utilization (buk, K00929; ptb, K00625,K00634) and complete modules like M00083 and M00086. Only two MAGs were predicted to encode genes for a complete Wood–Ljungdahl pathway, *Anaerolineales* sp. AS29adLBPA_61 (compl. 93.6%, cont. 3.01%) and *Deltaproteobacteria* sp. AS29adLBPA_53 (compl. 87.8% cont. 3.2%). Both are enriched in samples fed with butyrate, but *Deltaproteobacteria* sp. AS29adLBPA_53 has a stronger enrichment; however, these species remain at a quite low level in all the samples (maximum 0.16% and 0.37% relative abundance). Both these MAGs have also the ability to convert butyrate to Acetyl-CoA, which can enter the W-L pathway; however, the low relative abundance suggests that their impact on acetate conversion is limited.

#### Lactate

Analysis of the functional profile of MAGs enriched in the reactor fed with lactate revealed that five of them have a complete “SCFA and alcohol conversion: lactate D” pathway (Additional file 8). Despite these microbes being enriched in the reactor supplemented with lactate (Additional file 7), all these species have quite low relative abundance (Additional file 6). Among these, *Lachnospiraceae* sp. AS29adLBPA_171 (compl. 98.7%, cont. 1.5%) is interesting despite the low relative abundance (0.39%). This species encoded the electron transfer flavoprotein subunits A/B (K03521; K03522) (49957_15; 49957_16). The same pathway was described for the anaerobic lactate oxidation pathway used by the acetogen *Acetobacterium woodii* [17]. This enzyme complex works in a stable complex with FAD-dependent lactate dehydrogenase LDH, possibly encoded in the same transcriptional unit (49957_13; K00104, K03777). Additionally, the RNFA-G subunits were all identified in the genome and present in the same transcriptional unit (277210_85 to 277210_90); this complex can drive ferredoxin reduction with NADH as reductant. The other MAGs having high abundance belong to *Archaea* (e.g., *Methanocorpusculum* sp. AS29adLBPA_103; comp. 97.5%, cont. 4.9%, *Methanofollis liminatans* AS29adLBPA_137; comp. 96.8%, cont. 2.6%) or to species not having functional pathways directly related to lactate utilization (e.g., Verrucomicrobia sp. AS29adLBPA_71) (comp. 93.9%, cont. 2.03%). AS29adLBPA_103, AS29adLBPA_137 and AS29adLBPA_71 reached 6.4%, 3.4% and 1.05% relative abundance, respectively.

#### Propionate

Among the eight MAGs enriched in the samples fed with propionate, only *Deltaproteobacteria* sp. AS29adLBPA_60 (comp. 85.8%, cont. 1.9%) has a complete “SCFA and alcohol conversions: Propionate, pt 2” pathway (Additional file 8). This species has a low relative abundance (maximum 0.64%), and a manual verification of the gene content revealed that it harbors all the genes previously reported and belonging to the complex pathway involved in the conversion from propionate to acetate [96] (excluding succinyl-CoA synthetase, which absence could be due to limitations related to genome reconstruction). The conversion from propionate to succinyl-CoA is based on the path described in “KEGG propanoate metabolism”, followed by the conversion to pyruvate (KEGG TCA cycle) and finally to acetate (KEGG Carbon metabolism).

Other two dominant MAGs in the reactor fed with propionate are the unclassified *Candidatus* Cloacimonetes sp. AS29adLBPA_112 and the *Peptococcaceae* sp. AS29adLBPA_161. Both have a high-quality genome (compl. 98.9%, cont. 0%; compl. 94.3%, cont. 4.7%) and a maximum relative abundance close to 1%. As previously reported, the genomic analysis of the uncultivable “*Candidatus* Cloacamonas acidaminovorans” revealed the presence of all the genes involved in syntrophic propionate degradation. We can also suggest that *Cloacimonetes* sp. AS29adLBPA_112 have the same metabolism despite two genes in the pathway (succinate dehydrogenase and malate dehydrogenase) were not identified. Among the Archaea, *Methanospirillum* sp. AS29adLBPA_21 was enriched when propionate was the dominant substrate (Additional file 7). Previous data obtained from culture collections revealed that different isolated propionate‐degrading bacteria can act syntrophically with *Methanospirillum hungatei* [97].

#### Acetate

Acetate addition led to the enrichment of many *Archaea* (Additional file 7). Some of these microbes, for example *Methanosarcina mazei* AS29adLBPA_30 are well known for their ability to perform acetoclastic methanogenesis [98] some others, such as *Methanomassiliicoccaceae* sp. AS29adLBPA_85 (comp. 97.2%, cont. 5.9%, maximum relative abundance 2.4%) have complete pathways for the conversion of methanol to methane and a nearly complete pathway for the conversion of methylamines (Additional file 8). Since there are seven MAGs related to *Methanomassiliicoccaceae* having an increased abundance in the acetate-enriched medium, it is tempting to speculate that they use methanol generated by other microbes. It is known that acetogens such as *Acetobacterium woodii* can directly assimilate formate and methanol into the reductive acetyl-CoA pathway producing pyruvate [99]. Under the conditions of high acetate concentration they can possibly reverse the process leading to the generation of methanol. However, this process is still not demonstrated and can be an interesting target for future investigations. Notably, there was an enrichment of *Acetoanaerobium noterae* AS29adLBPA_56 (compl. 97.2%, cont. 3.8%) in the reactor fed with acetate, but it always remained at very low abundance. It was reported that this species can produce acetate from H_2_ and CO_2_ [100], and it has a nearly complete (83%) Wood–Ljungdahl pathway; however, the high acetate concentration present in the reactor suggests this is not the case. This species can possibly revert the Wood–Ljungdahl pathway using acetate to generate other sub-products.

The most abundant MAG identified among those enriched under these conditions is *Bacteroidales* AS29adLBPA_29 (relative abundance 2.8%), that, despite the high genome quality (compl. 93.2%, cont. 6.3%) does not have pathways related to acetate utilization (e.g., the Wood–Ljungdahl). The pathways involved in acetate utilization in this highly abundant species remain to be clarified.

## Discussion

### Effects of dominant products of acidogenesis: acetate, butyrate, lactate and propionate on methane formation

Using culture-independent techniques and a long-term system, we traced the processing of four dominant non-gaseous products of bacterial acidic fermentations to methane and carbon dioxide for better understanding of the metabolic pathways and syntrophic cooperation between microorganisms in the methane-yielding communities. Our system allows examining the acetogenic and methanogenic stages of anaerobic digestion and helps understanding microbial processes in multi-stage systems processing organic matter to biogas. However, anaerobic digestion of methanogenic sludge inside the bioreactor should also be considered.

The media subjected to methanogenesis were dominated by, or contained exclusively, one of the acidic fermentation products: lactate (M1), butyrate (M2), propionate (M3) or acetate (M4). Utilization of the substrate measured as % reduction of substrates COD revealed that acetate and lactate were used by the microbial communities more efficiently than butyrate and propionate, especially when the media contained exclusively one compound. With regard to the efficiency of methane production, the interpretation of the results is rather ambiguous. The highest methane production in Experiment 1 was achieved for butyrate processing with 60% substrate utilization; whereas in Experiment 2, for lactate processing with 74% substrate utilization. The tested substrates had a lower impact on the final performance of the bioreactors than expected. In contrast, large differences were observed in the results of isotopic analyses clearly showing that domination of acetotrophic or hydrogenotrophic pathways of methane synthesis is substrate dependent.

Acetate is a direct substrate for acetotrophic methanogens. It explains its efficient utilization and domination of the acetotrophic pathway of methanogenesis confirmed by isotopic analyses. Acetate detected in the effluents from bioreactor M4 in both experiments as well as from the other bioreactors could come from non-utilized substrate or from anaerobic digestion of methanogenic sludge inside the bioreactor. Acetate could also be oxidized to carbon dioxide and hydrogen. However, since acetate oxidation is an endoergic reaction (ΔG^0'^ =  + 94.9 kJ/reaction), it requires syntrophic cooperation with hydrogenotrophic methanogens, which results in ΔG^0'^ = − 36.3 kJ/reaction. Oxidation of butyrate and propionate are also endoergic reactions, with ΔG^0'^ =  + 48.3 kJ/reaction and ΔG^0'^ =  + 76.0 kJ/reaction, respectively, that become thermodynamically favorable with hydrogenotrophic methanogens, with ΔG^0'^ = − 17.3 kJ/reaction and ΔG^0'^ = − 22.4 kJ/reaction, respectively [101]. This explains the domination of hydrogenotrophic methanogenesis in M2 and M3 microbial communities revealed by isotopic analyses. Previously, we have concluded that lactate is oxidized mainly to acetate during the acetogenic step of AD and this includes the acetotrophic pathway of methanogenesis [18]. The present study confirms our previous results. Compared to butyrate and propionate, lactate is the most efficiently used substrate. It can be explained by the thermodynamics of lactate oxidation reactions, as it was discussed previously [18]. Briefly, lactate can be oxidized directly to acetate by many bacteria without the contribution of methanogenic *Archaea* according to the mechanism described for *Acetobacterium woodii* (ΔG^0'^ = − 61 kJ/mol) [17]. The fermentation of lactate to propionate is also a thermodynamically favorable reaction (ΔG^0'^ = − 169.7 kJ/reaction for *Desulfobulbus propionicus*) [102]. Propionate formation from lactate was also observed in this study. Formation of propionate probably induces the metabolic pathways of propionate oxidation. To summarize the results of the study, (i) butyrate and propionate are transformed mainly to acetate, while lactate is transformed mainly to acetate and propionate; (ii) oxidation of acetate and lactate determines the acetotrophic pathway of methanogenesis, whereas oxidation of butyrate and propionate determines the hydrogenotrophic pathway of methanogenesis. Propionate is one of the major intermediates in AD. It is estimated that 6–35% of methane can be produced from propionate. Propionate degradation to methane draws the attention of many researchers, because accumulation of propionate is often observed in bioreactors with poor methane production [22]. Interestingly, inhibition of methanogenesis in the bioreactor fed with a propionate-rich medium was not confirmed in this study. It could indicate that accumulation of propionate should be considered an indicator, rather than a cause of disturbances in anaerobic digestion and biogas production. Furthermore, accumulation of propionate is related to changes in the anaerobic digestion process, unstable pH and temperature, overload of feedstock, too high a concentration of short-chain fatty acids and hydrogen partial pressure, improper reactor configuration and hydraulic retention time [22, 103, 104].

### Lactate-, butyrate-, propionate- and acetate-selected microbial communities

Metagenomic analysis revealed that the dominant bacteria and archaea identified in this study are probably unknown, uncultivable species. A striking distinctive feature is the high number of *Archaea* found in the examined microbial communities, 31 archaeal species out of 234 microbial species identified (13.2%). A recent study performed metagenomic binning starting from a range of different biogas reactors but, out of 1635 microbial species identified, only 61 *Archaea* (3.7%) were recovered [37]. However, other studies revealed a remarkable number of archaeal species, which can also represent a large fraction of the entire microbiome [44, 105]. To determine how many MAGs reported in the present study represented new species not previously reported in the AD biogas database, a comprehensive Average Nucleotide Identity calculation was performed. Interestingly, 75.6% (177) of the MAGs identified here were not present in the global AD biogas database reported by Campanaro and colleagues, evidencing a very high level of new species recovered in this study. It can be speculated that the use of four diverse SCFAs as feedstock promoted the growth of species associated with the terminal part of the anaerobic degradation food chain (methanogenesis) where *Archaea* play a crucial role in methane production. On the other hand, most of the previous studies performed on the AD microbiome (those investigated by Campanaro and colleagues) did not focus on the acetogenic and methanogenic steps of the biogas food chain, where there are still many unknown microbes that were identified in the present project. As a confirmation of this finding, 20 out of 31 archaeal species identified here were not present in the global AD biogas database.

113 MAGs exhibited a comparable relative abundance in all bioreactors. They can be considered a sort of core microbiome with some dominant microbes such as *Methanothrix soehngenii*, *Methanoculleus* sp., representatives of *Bacteroidales*, *Spirochaetaceae* and unclassified bacteria. Interestingly, substrate-specific bacteria are not predominant in the microbial communities. Furthermore, the recognized acetate, lactate, butyrate, and propionate oxidizers described in the introduction are not in their majority present in the studied microbial communities form the bioreactors. The exceptions are butyrate-specific *Syntrophomonas wolfei* AS29adLBPA_159 (MQ) or lactate-specific *Desulfovibrio desulfuricans* AS29adLBPA_31. This is a confirmation that the vast majority of microorganisms, especially those requiring syntrophic growth and/or those having a very low growth rate, are yet to be isolated and cultivated. However, they represent the orders, families, and genera to which belong some recognized syntrophic bacteria involved in oxidation of products derived from acidic fermentations. These latter species were identified at high relative abundance in all bioreactors (e.g., *Spirochaetaceae* sp. AS29adLBPA_218, *Bacteroidales* sp. AS29adLBPA_33 or *Synergistales* sp. AS29adLBPA_149). Some other microbes were classified as “lactate-specific”, such as for example *Deltaproteobacteria* sp. AS29adLBPA_110 (MQ), *Firmicutes* sp. AS29adLBPA_210, *Clostridiales sp.* AS29adLBPA_49 (MQ); butyrate-specific *Syntrophomonas* sp. AS29adLBPA_81 (MQ), *Synergistales* sp. AS29adLBPA_149, *Syntrophaceae* sp. AS29adLBPA_197; propionate-specific *Peptococcaceae* sp. AS29adLBPA_161, *Desulfobacteraceae* sp. AS29adLBPA_204; acetate-specific *Clostridiales* sp. AS29adLBPA_6, *Sphaerochaeta* sp. AS29adLBPA_43 (MQ), *Geobacter* sp. AS29adLBPA_1 (MQ), *Anaerolineaceae* sp. AS29adLBPA_207.

Comments on substrate-specific and rare methanogenic species are reported in the Results section; additionally, functional analysis of medium-quality (MQ) MAGs has to be considered with caution, but their enrichment in specific samples can provide suggestions on their putative role.

Preference for specific substrates can be determined based on many different indicators, one being for sure the possibility to degrade a specific compound, but also syntrophic behaviors determined by compounds exchange between microbes are involved in shaping the structure of the microbiomes [51]. For this reason, it is expected that only a part of the “specialized microbes” is able to directly utilize the substrate provided; other species probably rely on the chemical compounds released. It can simply indicate a wide capability of one bacterium to use several different substrates, which should also be illustrated by investigation of genes expression (metatranscriptomics). Differences in genes expression seem to be indirectly supported by the results of stable carbon isotope composition of methane and carbon dioxide in the fermentation gas. Metagenomic analysis of the microbial communities clearly shows that dominant hydrogenotrophic or acetotrophic pathways of methane formation elucidated by isotopic analysis are not associated with changes in the contribution of methanogens utilizing hydrogen and carbon dioxide or acetate. The most dominant methanogens in all the bioreactors are *Methanothrix soehngenii* AS29adLBPA_138 (10–19%) and *Methanoculleus* AS29adLBPA_62 (5.5–11.8%), the latter being a Medium-Quality (MQ) MAG. *Methanoculleus,* similarly to other hydrogenotrophic methanogens, produced methane from carbon dioxide and hydrogen generated during syntrophic oxidation of SCFAs, this assumption being based on taxonomic assignment since the recovered genome of AS29adLBPA_62 was MQ and did not undergo functional analysis. It may indicate induction of genes of either the hydrogenotrophic or acetotrophic pathways of methane formation depending on the supplied substrate. Our explanation is as follows. Acetate as well as lactate, which is easily metabolized to acetate via the mechanism described for *A. woodii* [17], determines the acetoclastic pathway of methane formation in *Methanothrix soehngenii*. Butyrate and propionate initiate metabolic pathways found for methane-yielding microbial communities fed with ethanol, where the *Methanothrix* species reduced carbon dioxide to methane with electrons accepted via DIET [40]. This hypothesis should be confirmed by metatranscriptomic analysis of the microbial communities. Since, in addition to DNA, RNAs have been isolated from the same samples, the work on gene expression in the examined microbial communities is ongoing and the results will be included in a future report.

Recently, DIET is being increasingly mentioned in the context of anaerobic digestion [10, 106]. The microbial community fed with ethanol and dominated by the *Methanotrix* species exhibited an elevated abundance of the bacterial *pilA* gene and the methanogenic sludge showed a higher conductivity. In another study *Methanospirillum hungatei* was shown to form electrically conductive filaments that are analogs of e-pilli in *Geobacter* species. In the case of *M. hungatei,* it is the archaellum, whose core consists of tightly packed phenylalanines [107]. In our study the *Methanospirillum*_AS29adLBPA_21 strain was classified as a propionate-specific methanogen in the microbial community fed with the propionate-rich substrate.

In the study by Barua and co-workers (2018) [106], addition of conductive carbon fibers to the bioreactors fed with butyrate- and propionate-containing media resulted in (i) an increase of methane production, (ii) a higher efficiency of substrate utilization, (iii) an increased contribution of electroconductive bacteria such as *Desulfuromonas, Pseudomonas, Azonexus* or *Azovibrio* that accompanied butyrate and propionate oxidizers, and (iv) a domination of *Methanoseta* species among the methanogens. The results indicated that DIET is involved in processing of propionate and butyrate by the microbial community.

## Conclusions

In the present research, it was shown that the dominant components in the media (lactate, acetate, propionate or butyrate) subjected to methanogenesis moderately modified the final effect of bioreactor performance in terms of methane production and substrate utilization, whereas strongly affected the methanogenic pathways. Isotopic analysis evidenced different contributions of acetotrophic and hydrogenotrophic pathways for methane production, i.e., acetate and lactate favored the acetotrophic pathway, whereas propionate and butyrate favored the hydrogenotrophic pathway. Most of the 234 MAGs (31 archaeal and 203 bacterial species) were identified as new species. The core microbiome is represented by five MAGs present in high relative abundance (two methanogens: *Methanothrix soehngenii* and *Methanoculleus* sp., three bacterial MAGs classified only at high taxonomic level) and 108 other MAGs with a low relative abundance. Considering the relative abundance and/or their predicted functional role (determined according to KEGG pathways), three MAGs were found as propionate specific; four MAGs as lactate specific; four MAGs as butyrate-specific; and three MAGs as acetate-specific microbes. Analyzing the core microbiome and the substrate-specific species, we hypothesize the substrate may first of all change the metabolic activity of the bacteria/methanogens, rather than the composition of the microbial community. This requires confirmation using metatranscriptomic analysis.

Interestingly, we did not observe a reduction in methane production in the bioreactor fed with the propionate-rich medium. It may indicate that propionate commonly connected with inhibition of methanogenesis is rather an indicator than the cause of disturbances in anaerobic digestion. All these findings are relevant due to the fact that lactate, acetate, propionate and butyrate are the universal products of acidogenesis, regardless of feedstock.

## Supplementary Information

**Additional file 1. **Performance of bioreactors M1–M4: pH of effluents, substrate utilization and methane production—detailed data for Fig. 2.

**Additional file 2. **Medians of COD utilization and methane production during Experiments 1 and 2 (Table 1_Af2). Level of statistical significance for differences in medians (MWt) and distributions (KSt) between bioreactors performance over time during Experiments 1 and 2 (Table 2_Af2).

**Additional file 3. **Experimental data from individual measurements: δ ^13^C(CH_4_), δ ^13^C(CO_2_) and δ ^13^C_calc_.

**Additional file 4. **Evaluation of microbial composition based on analysis of unassembled reads performed independently from binning, using MetaPhlAn3.

**Additional file 5. **Quality of recovered MAGs according to results obtained from ckeckM software. Main parameters calculated for MAG genomes are also included.

**Additional file 6. **Taxonomic results obtained for recovered MAGs. Results obtained using CAT/BAT and GTDB-Tk software are reported.

**Additional file 7. **Relative abundance of MAGs calculated for different reactors and time points. MAGs enriched under different conditions are also reported.

**Additional file 8. **Functional annotation of recovered MAGs obtained using DRAM software (Distilled and Refined Annotation of Metabolism). KEGG IDs of annotated genes are also reported.

## Data Availability

All data generated or analyzed during this study are included in this published article [and its supplementary information files]. The datasets used and/or analyzed during the current study are available from the corresponding author on reasonable request.
