# Supplementary material for: Evaluation of acidogenesis products’ effect on biogas production performed with metagenomics and isotopic approaches
Source: Biotechnol Biofuels. 2021 May 29;14:125. doi: 10.1186/s13068-021-01968-0 (PMC8164749; doi:10.1186/s13068-021-01968-0)
Supplement: Supplementary file 2 — Additional file 2. Medians of COD utilization and methane production during Experiments 1 and 2 (Table 1_Af2). Level of statistical significance for differences in medians (MWt) and distributions (KSt) between bioreactors performance over time during Experiments 1 and 2 (Table 2_Af2). [file 13068_2021_1968_MOESM2_ESM.pdf]

Additional file 2

Table 1. Median of the COD utilization and methane production during Experiments 1 and 2.

Experiments	COD reduction [%]				Methane Production [dm^3/g COD reduced]			
	**M1**	**M2**	**M3**	**M4**	**M1**	**M2**	**M3**	**M4**
Experiment 1	75.5	68.1	69.3	74.5	0.44	0.59	0.43	0.45
Experiment 2	80.3	62.9	69.1	82.1	0.50	0.45	0.43	0.37

Table 2. The level of statistical significance for differences in median (MWt) and distributions (KSt) between the bioreactors performance over time during Experiments 1 and 2.

	COD reduction [%]						Methane Production [dm^3/g COD reduced]					
test	**M1/M2 p**	**M1/M3 p**	**M1/M4 p**	**M2/M3 p**	**M2/M4 p**	**M3/M4 p**	**M1/M2 p**	**M1/M3 p**	**M1/M4 p**	**M2/M3 p**	**M2/M4 p**	**M3/M4 p**
Experiment 1												
MWt	0.119	0.167	0.799	0.221	**0.005**	**0.013**	**0.026**	0.699	0.589	**0.015**	**0.002**	0.310
KSt	0.139	0.139	0.893	0.441	**0.005**	**0.031**	**0.031**	0.893	0.893	**0.031**	**0.005**	0.441
Experiment 2												
MWt	**0.000**	**0.000**	0.570	**0.025**	**0.000**	**0.001**	0.157	0.057	**0.009**	0.448	0.064	0.336
KSt	**0.000**	**0.000**	1.00	**0.015**	**0.001**	**0.001**	0.291	0.125	**0.015**	0.570	**0.046**	0.125

MWt – Mann-Whitney test, KSt – Kolmogorov-Smirnov test, p – level of statistical significance, bolded in read – statistically significant at ≤0.05
